# Pharmaco-psychiatry and gut microbiome: a systematic review of effects of psychotropic drugs for bipolar disorder

**DOI:** 10.1099/mic.0.001568

**Published:** 2025-06-18

**Authors:** Truong An Bui, Benjamin R. O’Croinin, Liz Dennett, Ian R. Winship, Andrew J. Greenshaw

**Affiliations:** 1 Neurochemical Research Unit, Department of Psychiatry, Faculty of Medicine and Dentistry, University of Alberta, Edmonton, Alberta, Canada; 2 Department of Medicine, Faculty of Medicine and Dentistry, University of Alberta, Edmonton, Alberta, Canada; 3 Geoffrey and Robyn Sperber Health Sciences Library, University of Alberta, Edmonton, Alberta, Canada

**Keywords:** antipsychotics, bipolar disorder, gastrointestinal microbiota/microbiome, mood stabilizers, psychotropics

## Abstract

Despite being one of the most common and debilitating mood disorders, bipolar disorder is often misdiagnosed and undertreated. Its pathogenesis is complex, with significant patient variability and inconsistent treatment effectiveness. The brain-gut-microbiota axis plays a critical role in bipolar disorder by modulating neurotransmitter secretion, gut peptides and systemic inflammation. However, the mechanisms by which psychotropic treatments influence gut microbiota composition and their implications for clinical outcomes remain poorly understood. This systematic review evaluated the impact of psychotropic drugs on gut microbiota and their potential role in bipolar disorder treatment outcomes. A comprehensive search across Ovid MEDLINE, Embase, APA PsycINFO, Scopus and PubMed yielded 314 articles, of which 12 met the inclusion criteria (last search: 13 August 2024). The studies included were those on adults with bipolar disorder type I or II receiving psychopharmacological treatments; those with group comparisons (e.g. healthy controls vs. medicated vs. non-medicated) investigating gut microbiome changes; and no restrictions applied to psychotic features, comorbid anxiety or prior treatment responses. Exclusions involved individual case reports, incomplete conference submissions or early terminated studies lacking efficacy analysis. Cochrane ROBINS-I V2 tool was used to measure the risk of bias, and the GRADE approach was utilized to rate the certainty of evidence in included studies. Two authors independently extracted data into Excel spreadsheets, categorizing demographic and clinical characteristics, describing microbiome analytic methods and summarizing findings on gut microbiome changes post-treatment. Given the high variability in methods and outcome measures across studies, all details were reported without data conversion. Data synthesis reveals that psychotropic treatments, including quetiapine and lithium, influence gut microbiota by increasing the abundance of beneficial bacteria supporting gut health and pathogenic bacteria linked to metabolic dysfunction. Notably, female patients exhibited more significant changes in microbial diversity following psychotropic treatment. Additionally, patients treated with psychotropics showed an increased prevalence of gut bacteria associated with multidrug antibiotic resistance. In bipolar patients treated with quetiapine, responders – those experiencing improved depressive symptom scores – displayed distinct gut microbiome profiles more closely resembling those of healthy individuals compared with non-responders. Responders also exhibited neural connectivity patterns similar to healthy subjects. These findings underscore the complex dual impact of psychotropic medications on gut microbiota, with potential consequences for both gut and mental health. While the enrichment of beneficial bacteria may support gut health, the rise in antibiotic-resistant and metabolically disruptive bacteria is concerning. Study limitations include methodological heterogeneity, inclusions of other psychiatric disorders, a high risk of bias in some studies due to incomplete statistical analyses or insufficient control for confounding factors and potential duplication of study populations arising from overlapping authorship. Further research is essential to elucidate the functional consequences of these microbial shifts and their influence on treatment efficacy. Nevertheless, this review highlights the potential of utilizing gut microbiota profiles to inform personalized treatment strategies, optimize therapeutic outcomes and minimize side effects in bipolar disorder. This study was registered with Open Science Framework (https://doi.org/10.17605/OSF.IO/3GUZR).

## Data Availability

The data supporting the findings of this study are available within the article and its supplementary materials. The protocol was registered with Open Science Framework (https://doi.org/10.17605/OSF.IO/3GUZR).

## Introduction

### Gut-brain axis crosstalk and mental illness: an overview

Current interest in a holistic view of nervous system function, including internal and external environmental modulators, now includes the bidirectional crosstalk between the gut and the brain, which is now referred to as the gut-brain axis. This review focuses on the relevance of the gut-brain axis to understanding mechanisms underlying mental illness, with a specific focus on the effects of drugs used to treat bipolar disorder (BP), a prominent and disabling mood disorder.

The gastrointestinal tract contains the largest, most abundant and diverse microbial reservoir in the human body, which serves as the first line of immune defence [1,3]. Gut microbiota comprises four major bacteria: *Bacteroidetes*, *Firmicutes*, *Proteobacteria* and *Actinobacteria* [4,6]. This microbiome can modulate the hypothalamic-pituitary-adrenal (HPA) axis, which mediates neuroendocrine and autonomic functions [7,8]. With digestion, the gut microbiome produces an abundance of neuroactive metabolites such as neurotransmitters and their precursors, including glutamate, noradrenaline, serotonin (5-HT), dopamine (DA), gamma-aminobutyric acid (GABA), tyramine, phenylethylamine and tryptamine that can regulate brain functions and behaviour [9,10]. These neurotransmitters play crucial roles in neurological and immunological activities in the brain [11]. Disturbed intestinal microbiota, often associated with reduced bacterial diversity, is associated with a variety of psychiatric diseases, including major depressive disorder (MDD) [12,14], BP [15] and schizophrenia (SCZ) [16]. Gut microbiome composition is especially sensitive to stressors in the central nervous system (CNS) that can activate the sympathetic nervous system and HPA axis, such as ageing, infection and injuries [17,18]. For instance, gut microbiota can influence brain-derived neurotrophic functions by altering the kynurenine pathway – the primary route for tryptophan metabolism, or by affecting the availability and impact of short-chain fatty acids (SCFAs) in the brain [19].

Serotonin transporters (SERTs) are distributed across the brain and located on the epithelial cells of the intestinal mucosa, where they facilitate the removal of 5-HT from the interstitial space subsequent to its release by enterochromaffin cells [20,22]. 5-HT plays a significant role in regulating intestinal permeability; therefore, it is reasonable to hypothesize that ileal permeability may be influenced by the direct effects of the psychotropics on SERT [23,25]. Stress and depression are known to increase gut barrier permeability and cause epithelial barrier defects, or ‘leaky gut,’ which results in gut bacteria, such as *Escherichia coli* and their LPS, translocating into circulatory fluids within the body and evoking an inflammatory response [15,26, 27]. These can further promote the occurrence and progression of psychiatric disorders [28,29]. However, mechanisms for these relationships have yet to be identified [30].

### BP epidemiology and pathophysiology

BP is the 17th leading contributor to the global burden of disease, just after MDD, anxiety disorders, SCZ and dysthymia, affecting >1% of the worldwide population [31,32]. In 2019, BP was reported to account for 8.5 million global disability-adjusted life-years [33]. The global economic burdens and healthcare costs of BP are enormous, as they affect not only the patients but also family members and caregivers [34,36].

BP is characterized by biphasic mood episodes of mania (or hypomania) and depression, which result in extreme and unpredictable emotion, thought and energy disturbances [37,38]. Mania can manifest as hyperactivity, irritability, grandiosity, decreased need for sleep, increased talkativeness, distractibility, delusions, flight of ideas, psychomotor agitation, risk-taking behaviour and highly elevated mood [39,40]. In contrast, depressive episodes are characterized by sadness, reduced interest or pleasure, fatigue, social withdrawal, low self-esteem, psychomotor retardation or agitation, sadness, insomnia or hypersomnia and change in appetite and weight [40,41]. Diagnoses of BP in the Diagnostic and Statistical Manual of Mental Disorders (DSM-V-TR) include bipolar I disorder (BP-I), bipolar II disorder (BP-II), cyclothymic disorder and substance/medication-induced BP [42].

Medications are generally the most critical component of BP treatment [43,44]. Pharmacological therapies used for BP can be divided into mood stabilizers, antipsychotics and anticonvulsants (also known as antiepileptics) [45,46]. Selective serotonin reuptake inhibitors (SSRIs) are also often used ‘off-label’ to manage depressive episodes; however, they have the risk of inducing mania, which warrants serious caution [47]. However, this issue remains contentious, as there is conflicting evidence regarding cycle acceleration in BP with modern antidepressants, particularly when used alongside mood stabilizers [47,50].

During acute treatment, a mood state (e.g. depression) may transition or spill over to its opposite (e.g. mania), adding complexity to clinical decisions regarding psychotropic selection and dosing [51,53]. This mood switch process is a distinctive feature of BP compared with other psychiatric conditions, presenting a unique challenge in its management [54]. Additionally, among patients receiving pharmacotherapy, there is up to 35% relapse within the first year in adults, and the risk of relapse rises to 65% after 4–5 years [43,44, 55, 56].

Functional imaging, such as functional magnetic resonance imaging (fMRI) and positron emission tomography (PET), offers a powerful means of investigating the interplay between the gut microbiome and brain activity in BP. Imaging can identify biomarkers that differentiate BP from other psychiatric conditions, such as unipolar depression or SCZ, which often present overlapping symptoms [57]. It also allows researchers to assess the impact of pharmacological treatments (e.g. mood stabilizers) and psychotherapy on brain function, providing insights into therapeutic mechanisms, predicting treatment outcomes response and creating tailored interventions [58,60].

Techniques like PET enable the study of neurotransmitter systems, such as DA and 5-HT, which are implicated in the pathophysiology of BP [61,63]. fMRI can identify abnormal connectivity in brain networks like the default mode network (DMN), prefrontal cortex, hippocampus and amygdala. These networks govern self-referential thinking and emotional processing and are disrupted in BP [64,67]. For example, compared with healthy controls (HCs), BP patients have greater functional connectivity (FC) between the salience and DMN [68] and altered activation within the DMN [64,66, 69].

5-HT is a pivotal neurotransmitter in mood stabilization, and its dysregulation plays a significant role in the manic and depressive symptoms of BP [70,72]. Dysregulation of 5-HT metabolism has been associated with intestinal microbiota-induced inflammation [73,76], and these neurotransmitter abnormalities can be detected through functional imaging techniques such as fMRI and PET [77,79]. Previous studies showed that gut dysbiosis can divert tryptophan, the precursor for 5-HT synthesis, into the kynurenine pathway, thereby reducing 5-HT availability while increasing neurotoxic metabolites [80,82]. Additionally, SCFAs produced by gut bacteria can affect the SERT expression and 5-HT receptor sensitivity, which regulates 5-HT reuptake [83]. Functional imaging can track changes in glucose metabolism in brain regions affected by gut-derived metabolites, offering a direct visualization of how gut health shapes brain energy utilization [84,85].

Psychotropic agents used to treat BP patients are known to induce weight gain and other metabolic disorders such as obesity, insulin resistance, dyslipidaemia, hypertension, impaired glucose tolerance and high rates of atherosclerotic disease [86,95]. These side effects suggest a connection between BP pathophysiology/treatment and metabolism, as well as the gut microbiome. Thus, a comprehensive understanding of the interactions between the gut microbiome, pharmacotherapy and treatment outcomes is vital. Although the relationship between BP and the gut microbiome is still relatively unexplored, available evidence describing differences in gut microbiome composition between BP and HC has been summarized fairly recently [96,99]. To our knowledge, this is the first systematic review of the potential effects of psychotropics on the microbiome of treated and untreated BP individuals.

### Objectives

As the first systematic review on the topic, this review investigates how pharmacological treatments might modulate inflammatory processes, metabolic functions and neurotransmitter signalling pathways within the gut-brain axis in BP. Gut microbiome in psychiatric disorders is a very complex and rapidly evolving field, and BP is a heterogeneous disorder, with individuals often experiencing varying responses to pharmacological treatments. BP patients also frequently develop comorbidities, including metabolic syndrome, gastrointestinal issues and other psychiatric conditions. Exploring the role of the gut-brain axis in the context of BP pharmacotherapy provides a novel lens through which these discrepancies and complexities might be explained and lead to more holistic approaches to managing BP. Moreover, understanding these connections could also pave the way for novel personalized therapeutic strategies, inspiring further studies and clinical trials focused on microbiome-targeted interventions.

## Methods

### Inclusion criteria

This review followed the Preferred Reporting Items for Systematic Reviews and Meta-Analyses (PRISMA) guidelines, and the PRISMA 2020 checklist was used [100]. The PRISMA 2020 checklist and abstract checklist are available in Tables S3 and S4 (available in the online Supplementary Material). The protocol for the proposed study is registered with Open Science Framework (ID: 3GUZR; registration DOI: https://doi.org/10.17605/OSF.IO/3GUZR).

The inclusion criteria for studies were as follows: (1) studies of adults diagnosed with BP-I or BP-II, (2) participants received psychopharmacological BP intervention (e.g. mood stabilizers, antipsychotics and antidepressants), (3) studies must have group comparisons, such as medicated participants vs. placebo/HCs/unmedicated participants and (4) studies must investigate the changes in the gut microbiome with no limitations on experimental design (e.g. longitudinal or cross-sectional). There were no limitations regarding the existence of psychotic features, the presence of comorbid anxiety symptoms or disorders or inadequate responses to prior treatments.

The exclusion criteria were as follows: (1) individual case reports relating to medications, (2) conference submissions that lack complete quantitative or qualitative reports, and (3) studies that terminated early without efficacy analysis.

### Data collection and search strategy

Strategies to identify eligible studies included a systematic search of five electronic databases: Ovid MEDLINE, Embase (via Ovid), APA PsycINFO (via Ovid), PubMed and Scopus, up to 13 August 2024. The search was conducted with assistance from a library and information science specialist (LD) to ensure the inclusion of all relevant articles. The search included three concepts combined with Boolean AND: BP, gut microbiome and mood-stabilizing drugs. Each concept included subject headings and synonyms combined with Boolean OR and was optimized for each database. No methodological, publication, language or date limits were added to the search. The full search strategy is available in Table S1.

**Fig. 1.**
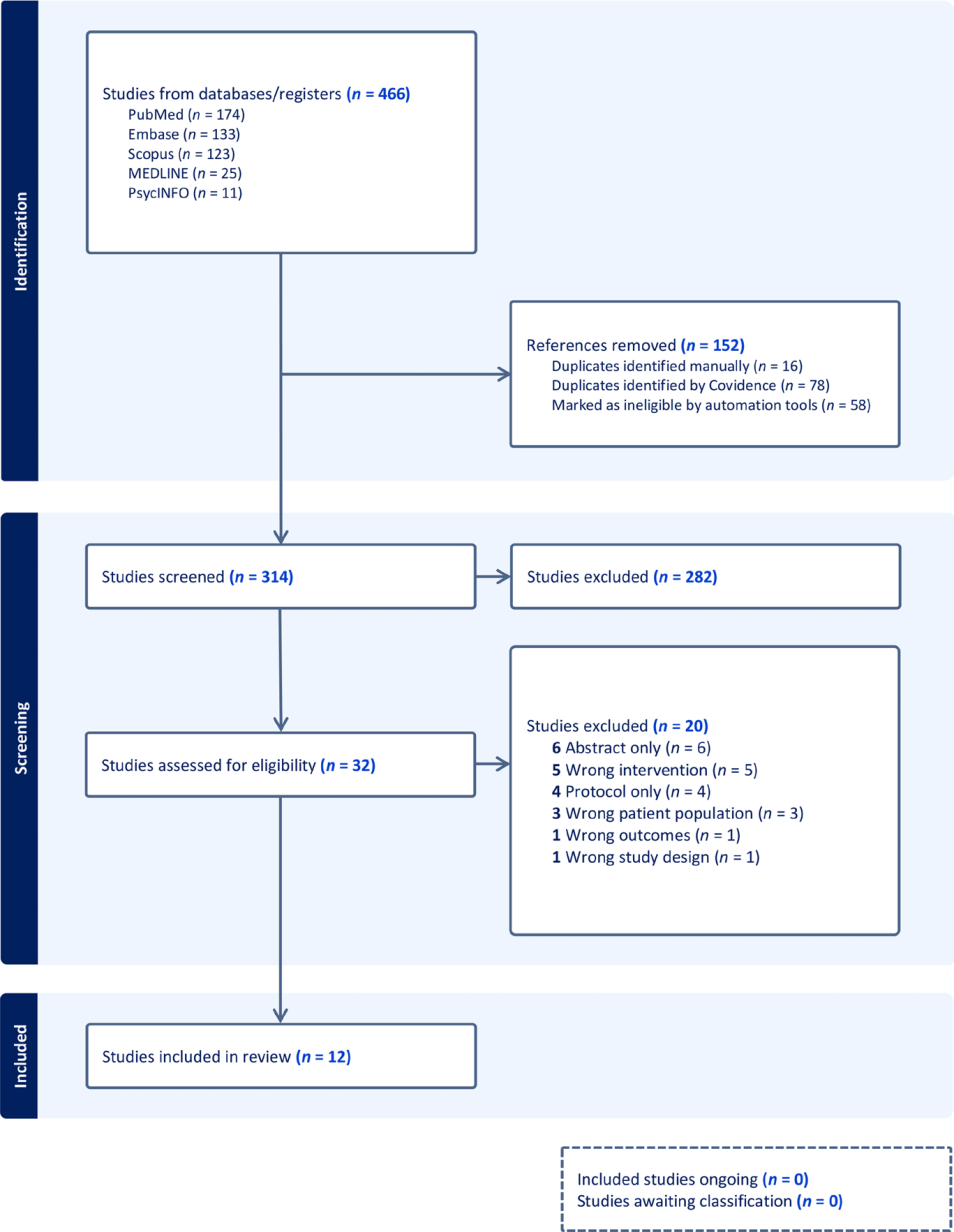
Study selection with PRISMA flow diagram.

All citations were reviewed by title, then by abstract and then by full text and were screened according to the inclusion and exclusion criteria by two reviewers independently (TAB and BRO) using the Covidence tool [101]. When there was uncertainty regarding the inclusion of an article or disagreement, the final decision was made by AJG. A total of 12 full-text articles were selected (Fig. 1).

### Data extraction

Two authors (TAB and BRO) systematically extracted the following information from the included articles into an Excel spreadsheet. Data were extracted in the following categories: (1) demographic and clinical characteristics of the study samples, including sample size, age range, sex ratios, body mass index (BMI), diagnostic group, medications, inclusion criteria and exclusion criteria; (2) microbiome analytic methodology includes gut genetic quantification, gut diversity measures, brain imaging (in selected studies) and covariates’ effects; and (3) study findings, including group differences in taxonomic abundance and correlations between medications and associated gut microbiome characteristic changes. The primary outcomes from these studies were response to treatment and all-cause discontinuation (e.g. withdrawal consent). Since the methods and outcome measures in each study vary greatly from one another, we reported these differences and all details and did not have to do any data conversion.

### Risk of bias and certainty of evidence assessment

**Fig. 2.**
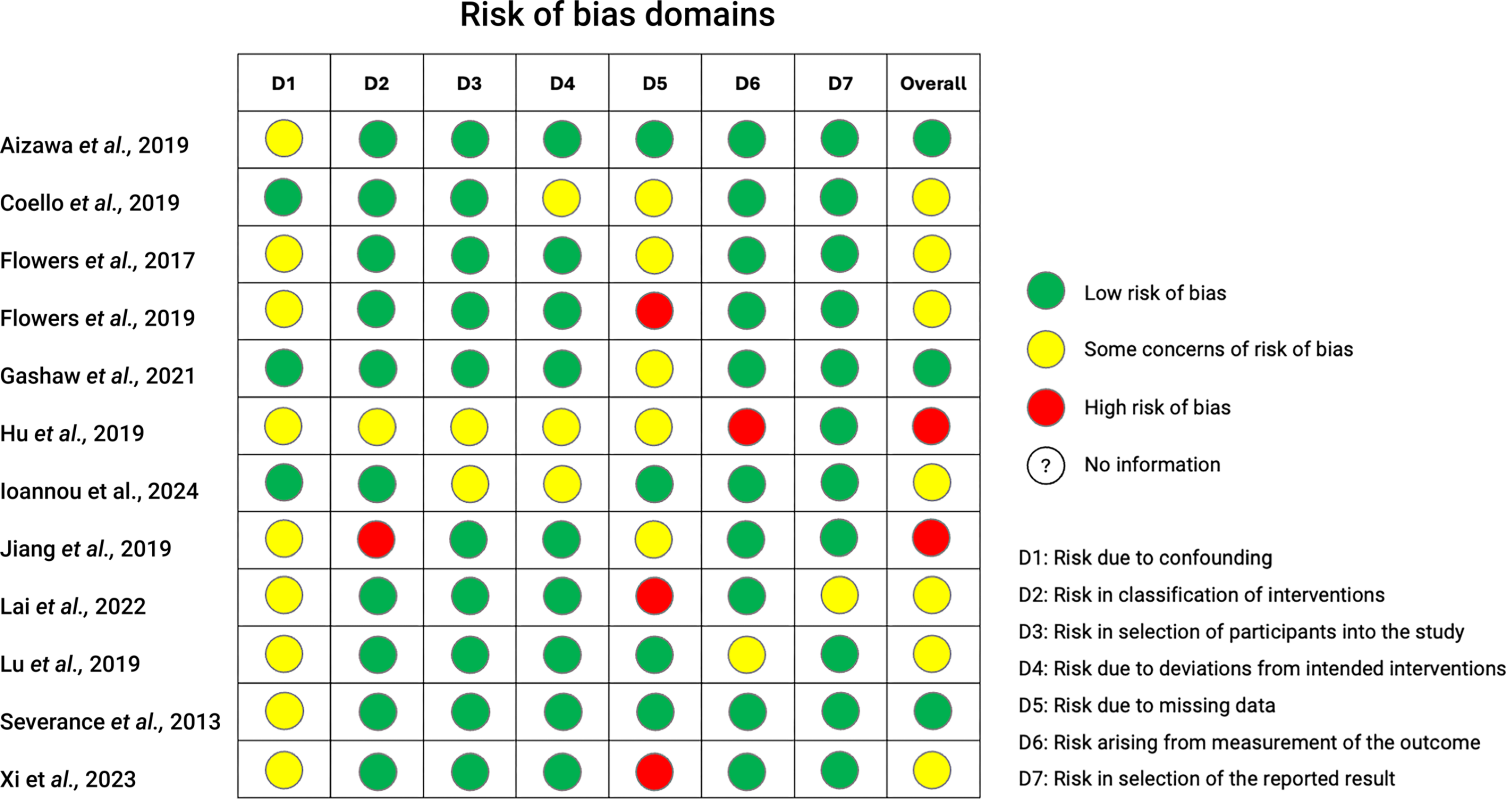
Risk of bias assessment.

Given the diverse study designs in our systematic review, the risk of bias in included studies was evaluated using the Cochrane risk of bias ROBINS-I V2 tool, which is suitable for cross-sectional interventional and longitudinal interventional studies [102]. Two reviewers (TAB and BRO) independently assessed the studies for potential biases, including selection and reporting biases, and resolved discrepancies through discussion (Fig. 2).

The certainty of the evidence was assessed using the GRADE approach. This method evaluated the study design, findings’ consistency, evidence’s directness and effect estimates’ precision. The assessment provided an overall confidence rating for each outcome, guiding the interpretation of results and conclusions. A summary of findings table was created using the GRADE Working Group’s software GRADEpro GDT (Fig. S1).

## Results

The search strategy is provided in Table S1. Fig. 1 illustrates the literature search process. Database searches identified 466 references; 314 articles were identified from all databases after removing duplicates. Of these, 282 were excluded based on the title and abstract and 20 based on the full text, resulting in 12 articles for inclusion (Table 1).

**Table 1.** Studies included for systematic review

Author	Name
Aizawalet al.^ 2019 ^[134]	*Bifidobacterium* and *Lactobacillus* counts in the gut microbiota of patients with BP and HCs
Coelloet al.^ 2019 ^[105]	Gut microbiota composition in patients with newly diagnosed BP and their unaffected first-degree relatives
Flowerset al.^ 2017 ^[113]	Interaction between atypical antipsychotics and the gut microbiome in a bipolar disease cohort
Flowerset al.^ 2019 ^[111]	Effects of atypical antipsychotic treatment and resistant starch supplementation on gut microbiome composition in a cohort of patients with BP or SCZ
Gashawet al.^ 2021 ^[107]	Assessment of gut bacteria profile and antibiotic resistance pattern among psychotropic drug users: comparative cross-sectional study
Huet al.^ 2019 ^[112]	Gut microbiota changes in patients with bipolar depression
Ioannouet al.^ 2024 ^[108]	Reproducible gut microbial signatures in bipolar and SCZ spectrum disorders: a metagenome-wide study
Jianget al.^ 2019 ^[106]	The microbiome in bipolar depression: a longitudinal study of one pair of monozygotic twins
Laiet al.^ 2022 ^[103]	Metagenomic analysis reveals gut bacterial signatures for diagnosis and treatment outcome prediction in bipolar depression
Luet al.^ 2019 ^[110]	Gut microbiota in bipolar depression and its relationship to brain function: an advanced exploration
Severanceet al. 2013 [109]	Discordant patterns of bacterial translocation markers and implications for innate immune imbalances in SCZ
Xiet al. 2023 [104]	Brain-gut microbiota multimodal predictive model in patients with bipolar depression

### Overview of study characteristics

Demographic and clinical characteristics of the study samples are presented in detail in Tables2  3. The study with the largest sample was conducted in Baltimore, USA (*N*=294), and the remaining study samples ranged from *N*=6 to *N*=231. All studies were conducted between 2013 and 2024. Location varied widely between studies, including the USA, China, Denmark, Ethiopia, Japan and the Netherlands, and most patients were recruited from local psychiatric hospitals. In some studies, a variety of medication combinations was used, such as antidepressants and benzodiazepines, further complicating the results. Most studies (10/12) include HCs (Table 2). In two studies, BP patients were further divided into quetiapine-responded and non-responded patients based on whether the treatment reduced HAMD-17 score by 50% compared with baseline and a random forest classification model [103,104]. Two studies also recruited unaffected relatives (URs) and BP patients [105,106]. Three studies also included patients with SCZ spectrum disorder (SSD) [107,109].

**Table 2.** Demographic and clinical characteristics of included studies

Study	Sample size*	Location	Design	Sample type	Diagnosis	Response	Medication (*N*)
Aizawa *et al*., 2019 [134]	*N*=122HC=58 (♀ 36; ♂ 22)BP=39 (♀ 22; ♂ 17)AAP=13LL=29	Tokyo, Japan	Cross-sectional observational cohort	Inpatientcommunity	BP=39BP-I=13BP-II=26	n/a	***AAP:*** sodium valproate (8), carbamazepine (4)***Antidepressant*** (12)***Lithium*** (16)***Lamotrigine*** (13)
Coello *et al*., 2019 [105]	*N*=229HC=77 (♀ 47; ♂ 30)UR=39 (♀ 21; ♂ 18)BP=113 (♀ 70; ♂ 43)AAP=43LL=44	Copenhagen, Denmark	Cross-sectional observational cohort	Community	BP-I=44BP-II=65BP NOS=4	n/a	***AAP*** (43)***Antidepressants*** (30)***Antiepileptics*** (58)***Lithium*** (44)
Flowers *et al*., 2017 [113]	*N*=117†AAP=49 (♀ 34; ♂ 12)NAAP=68 (♀ 48; ♂ 21)LL=31	MI, USA	Cross-sectional observational cohort	Community	BP=117	n/a	***AAP:*** clozapine, olanzapine, risperidone, quetiapine, asenapine, ziprasidone, lurasidone, aripiprazole, paliperidone, iloperidone***Antidepressants*** (52): bupropion, venlafaxine, sertraline, duloxetine, fluoxetine, citalopram, escitalopram***Mood stabilizers*** (60): topiramate, phenobarbital, lamotrigine, gabapentin, divalproex sodium, carbamazepine, lithium***Benzodiazepines*** (32): lorazepam, alprazolam, temazepam, clonazepam, diazepam
Flowers *et al*., 2019 [111]	*N*=37AAP=21 (♀ 9; ♂ 12)LL=16 (♀ 7; ♂ 9)	MI, USA	Cross-sectional interventional longitudinal cohort	Community	*AAP group:*BP-I=9BP-II=3SZA=5SCZ=4*LL group:*BP-I=10BP-II=6	n/a	***AAP*** (3 weeks): clozapine, olanzapine, risperidone, quetiapine, ziprasidone+starch (2 weeks)***Mood stabilizer*** (3 weeks): lithium, lamotrigine***Concomitant medications*:** antidepressants, benzodiazepines, medications for hypertension, diabetes mellitus and hyperlipidaemia
Gashaw *et al*., 2021[107]	*N*=214†HC=107 (♀ 27; ♂ 80)SCZ=57BP=28Depression=21	Jimma, Ethiopia	Cross-sectional observational cohort	Inpatientcommunity	SCZ=57BP=28Depression=21	n/a	***Antipsychotics*** (54)***Mood stabilizers*** (14)***Antidepressants*** (11)***Combination therapy*** (28)
Hu *et al*., 2019[112]	*N*=97HC=45 (♀ 22; ♂ 23)BP=52 (♀ 25; ♂ 27)	Zhejiang, China	Cross-sectional interventional longitudinal cohort	Inpatientcommunity	BP-I=12BP-II=38BP NOS=2	NBP=5‡RBP=12	***Quetiapine*** (4 weeks)Maintenance dose: 200–300 mg day^−1^
Ioannou *et al*., 2024 [108]	*N*=231HC=128 (♀ 77; ♂ 51)BP=66 (♀ 48; ♂ 18)AAP=34LL=35NAAP=35SSD=37 (♀ 16; ♂ 21)AAP=28LL=3NAAP=1 5	Groningen, Netherlands	Cross-sectional observational cohort	Inpatientcommunity	BP=66SCZ/SZA=37	n/a	***Antipsychotics*** (62)***Lithium*** (38)***Antidepressants*** (29)***Anticonvulsants*** (23)
Jiang *et al*., 2019 [106]	*N*=6HC=4 (♀ 2; ♂ 2)UR=1 (♀ 1)BP=1 (♀ 1)	Zhejiang, China	Longitudinal case-control	Inpatientcommunity	BP=1	n/a	***Lamotrigine***Maintenance dose: 100 mg day^−1^***Quetiapine***Maintenance dose: 50 mg day^−1^
Lai *et al*., 2022 [103]	*N*=122HC=60 (♀ 31; ♂ 29)BP=62 (♀ 23; ♂ 39)	Zhejiang, China	Cross-sectional interventional longitudinal cohort	Inpatientcommunity	BP-I=12BP-II=45BP NOS=5	NBP=19‡RBP=35Other=9	***Quetiapine*** (4 weeks)Initial dose: 50 mg day^−1^Maintenance dose: 200–300 mg day^−1^
Lu *et al*., 2019 [110]	*N*=63HC=27 (♀ 12; ♂ 15)BP=36 (♀ 15; ♂ 21)	Zhejiang, China	Cross-sectional interventional longitudinal cohortRCT	Inpatientcommunity	BP-I=10BP-II=26	n/a	***Quetiapine*** (4 weeks)Initial dose: 50 mg day^−1^Maintenance dose: 300 mg day^−1^***Propranolol***: 10–20 mg***Glycerine enema***
Severance *et al*., 2013 [109]	*N*=294HC=78 (♀ 56; ♂ 22)BP=75 (♀ 52; ♂ 23)AAP=57NAAP=18SCZ=141 (♀ 56; ♂ 85)	Baltimore, US.Cologne, Germany	Cross-sectional observational cohort	n/a	BP=75SCZ=141	n/a	***Antipsychotics***SCZ=141BP=57
Xi *et al*., 2023[104]	*N*=142HC=39 (♀ 23; ♂ 16)BP=103QTP=75 (♀ 38; ♂ 37)	Zhejiang, China	Cross-sectional interventional longitudinal cohort	Inpatientcommunity	BP=103	RBP=43‡NBP=32	***Quetiapine*** (4 weeks)Maintenance dose: 200–300 mg day^−1^

*Participants included in the final data analysis.

†There was inconsistency in the total number of participants reported and the breakdown of participants by sex.

‡Based on the degree of improvement in depressive severity post-treatment (HAMD change ≥50 %), patients were classified into responder and non-responder groups.

AAP, atypical antipsychotics; LL, lithium- or lamotrigine-treated group; N/A, Not applicable; NAPP, non-atypical antipsychotics; NBP, quetiapine non-responded patients; NOS, not otherwise specified; QTP, quetiapine; RBP, quetiapine responded patients; RCT, registered clinical trial; SZA, schizoaffective disorder; UR, unaffected first-degree relative.

**Table 3.** Study criteria and analysis methodology of included studies

Study	Study criterion		Analysis method		
	Inclusion criterion*	Exclusion criterion*	Analysis type	BP diagnosis	Covariate
Aizawa *et al*., 2019 [134]	Diagnosed BP from the outpatient clinic of the National Center of Neurology and Psychiatry	Prior medical history of CNS disease, including epilepsy and severe head injury, substance abuse or dependence, mental retardation, recent use of antibiotics, history of gastrointestinal surgery, severe congenital abnormalities or any severe medical conditions	16S or 23S rRNA targeted RT-qPCRRadioimmunoassayANCOVA	MINIDSM-IVYMRSHAMD-17	AgeSexBMIAge of onsetComorbiditiesEducationMedication
Coello *et al*., 2019 [105]	Newly diagnosed or first episode BP from the Copenhagen Affective Disorder Clinic	n/a	16S rRNA sequencingPCRImmunoturbidimetric assayPERMANOVA	DSM-VSCANICD-10HAMD-17YMRSIPAQ	AgeSexSmokingWaist circumferencePhysical activityMedicationIllness duration
Flowers *et al*., 2017 [113]	Diagnosed BP from the Prechter Longitudinal Study of Bipolar Disorder	n/a	16S rRNA sequencingPCRAMOVAInverse Simpson diversity indexLEfSeLDA	DIGSHDRSYMRSASRMPHQ-9	AgeSexBMIMedication
Flowers *et al*., 2019 [111]	BP-I, BP-II or BP NOSBP with psychosisSCZ or SZABP treated with an AAP – clozapine, olanzapine, risperidone, quetiapine or ziprasidone – or lithium and/or lamotrigine for at least 6 months	Unable to give informed consentHospitalization or antimicrobial exposure within 6 months; receipt of systemic corticosteroids, immunosuppressant drugs, cytotoxic agents or hormonal contraceptives; presence of any serious medical condition that would significantly affect weight changes; uncontrolled gastrointestinal disorders; chronic heavy alcohol consumption; or recent unstable dietary history	16S rRNA sequencingPERMANOVAPERMDISPLEfSeASA24SF-36	DSM-IV	AgeSexRaceEducationBMINutritionHospitalizationMetabolic syndromePhysical activitySmokingMedication
Gashaw *et al*., 2021 [107]	Psychiatry patients from Jimma Medical Centre psychiatric clinic	Antibiotic use within the last 6 months of the data collection	Antimicrobial susceptibility testing	n/a	AgeSexSocio-demographicMedicationIllness duration
Hu *et al*., 2019 [112]	Diagnosed BP with a current depressive episode from Psychiatric Department of First Affiliated HospitalHDRS-17 score ≥14First episode or psychotropic drug free for at least 3 months	Comorbid psychiatric disordersSevere physical diseasesAcute/chronic infectionsSubstance abusePregnancyBreastfeedingAntibiotic, probiotic or prebiotic use for less than 4 weeks before study	16S rRNA sequencingPERMOVALEfSePCoAPCR	DSMM-IV-TRHDRS-17MADRSYMRSMINI	AgeSexBMIAge of onsetDuration of illnessIllness durationMedication
Ioannou *et al*., 2024 [108]	BP and SSD patients from the Department of Psychiatry of the University Medical Center Groningen	Pregnancy or breastfeedingActive liver, kidney or pancreatic disease, other clinically significant or unstable medical problem, including inflammatory bowel disorder (IBP), short bowel syndrome or acute/chronic pancreatitis	Metagenomic shotgun sequencingPCoAPERMOVAOLS	DSMMINI	AgeSexBMIDietBPRSSmokingEducationIncomeMedication
Jiang *et al*., 2019 [106]	Hospitalized BP	n/a	16S rRNA sequencingPCRLEfSePCoA	HDRS	AgeSexBMIEducationalPhysical activityDietAlcohol consumptionSmokingAntibioticsMedication
Lai *et al*., 2012 [103]	Diagnosed BPHDRS-24≥14Drug-naïvePsychotropic substances-free for at least 3 monthsMADRS ≤2Absence of apparent suicide thoughts or past attempted suicide	Chronic infection, severe systemic diseases, autoimmune diseasesAntibiotic, prebiotics, probiotics within 4 weeks before screeningPregnancy, lactation, menstruation femalesComorbid psychiatric disordersHistory of cranial traumaContraindications for MRI	Metagenomic sequencing rs-fMRIPERMANOVALEfSe	DSM-IV-TRYMRSHAMAHDRS-24MADRSMINI	AgeSexBMIEducationAge of onsetIllness durationSocio-demographic
Lu *et al*., 2019 [110]	Diagnosed BP from inpatient/outpatient department of the First Affiliated HospitalNo treatments or medications stopped for more than 3 months	Gastrointestinal disease, infectious disease, fever, other physical diseasesAutoimmune disease, endocrine disease, other mental disordersAntibiotics, probiotics, prebiotics, synbiotics or yoghurt within a monthTaking lactulose, prokinetic drugs or antibiotic treatment in the last monthHistory of substance abuse (nicotine dependence, caffeine use and others)	MADRSYMRSNIRS measurementT lymphocyte flow cytometry qPCR	SCIDDSM-IV-TRMADRSYMRSMINI	AgeSexMarriageBMIEducationAge of onsetIllness duration
Severance *et al*., 2013 [109]	Diagnosed BP and SCZAge 18–65 years old	Substance abuse/dependenceMental retardation or medical disorders affecting cognitive performance	ELISA (sCD14, LBP)	DSM-IV	AgeSexBMIRaceSmoking
Xi *et al*., 2023 [104]	Diagnosed BP depressed from Psychiatric Department of First Affiliated HospitalHAMD-17≥14First episode or free from psychotropics for at least 3 monthsNo probiotic preparation for at least 1 monthNo history of respiratory, urinary or digestive infections	Psychiatric comorbidities and cognitive impairmentsSerious suicidal ideation or behaviour, or intense euphoria, substance abuse (alcohol, narcotics, etc.)Severe physical sickness, intestinal diseasePregnancy or breastfeeding women	Metagenomic sequencingPCR rs-fMRIPCoA	DSM-IV-TRHAMD-17YMRSMINI	AgeSexBMISmokingHandednessHAMD

*For BP/SCZ patients.

AMOVA, Analysis of Molecular Variance ; ASA24, Automated Self-Administered 24-Hour Dietary Assessment Tool; ASRM, Altman Self-Rating Mania Scale; BP, Bipolar disorder; BPRS, Brief Psychiatric Rating Scale; DIGS, Diagnostic Interview for Genetic Studies; ELISA, Enzyme-linked Immunosorbent Assay; HAMA, Hamilton Anxiety Rating Scale; HAMD, Hamilton Depression Rating Scale; HDRS-24, 24-item Hamilton Depression Rating Scale; IPAQ, International Physical Activity Questionnaire; LDA, Linear Discriminant Analysis; LEfSe, Linear Effect Size analysis; MADRS, Montgomery-Asberg Depression Rating Scale; MINI, Mini International Neuropsychiatric Interview; MRI, Magnetic Resonance Imaging; NIRS, Near-Infrared Spectroscopy; OLS, Ordinary Least Squares linear regression; PCoA, Principal Coordinates Analysis; PERMDISP, Permutational Multivariate Analysis of Dispersion; PERMOVA, Permutational ANOVA; PHQ-9, Patient Health Questionnaire-9; qPCR, quantitative PCR; rs-fMRI, resting-state Magnetic Resonance Imaging; SCAN, Schedules for Clinical Assessment in Neuropsychiatry; SCID, Structured Clinical Interview for DSM-IV-TR disorders; SCZ, Schizophrenia; SF-36, 36-Item Short-Form Health Survey; SSD, Schizophrenia Spectrum Disorder; YMRS, Young Mania Rating Scale.

### Study design

Most studies employed a cross-sectional cohort design. There was significant heterogeneity between studies; among the 12 articles, 1 was a registered clinical trial [110] and 6 were longitudinal studies [103,104, 106, 110,112]; thus, only a subset shared comparability. Three studies specified a priori taxa for study, as opposed to studying the entire gut microbiota [107,109, 110]. Four studies (4/12) were interventional, where participants underwent a 3–4-week treatment with psychotropic medications [103,104, 110, 111]. These studies employed a fixed dose of quetiapine (300 mg day^−1^) without a discernible dose-response correlation. The remaining eight studies were observational, where researchers monitored and compared the effects of psychotropic medications between individuals who were taking psychotropics and those who did not.

Potential confounds when studying the microbiome, including age, sex, BMI and medications, were all recorded. BP patients were recruited and diagnosed using various means, including admission in the hospital’s inpatient/outpatient unit, using DSM-IV or DSM-V, Young Mania Rating Scale (YMRS), Hamilton Anxiety Rating Scale (HAMA), Hamilton Depression Rating Scale (HDRS), Montgomery-Asberg Depression Rating Scale (MADRS), Mini International Neuropsychiatric Interview (MINI) and Diagnostic Interview for Genetic Studies (DIGS). Most studies excluded patients with substance abuse, cognitive impairments, intellectual disabilities, pregnancy, severe physical disabilities and uses of antibiotics, probiotics and/or prebiotics within 4 weeks of screening. A variety of sequencing methods (metagenomic sequencing, 16S RNA sequencing and quantitative PCR) and analysis pipelines (permutational analysis of variance, analysis of molecular variance, linear effect size analysis and principal coordinate analysis) were used to characterize microbiome composition (Table 3).

### Quality assessment results

The quality assessment results are reported in Fig. 2 and Table S2. ROBINS-I V2 tool was used to evaluate the risk of bias for all included studies, which examines seven bias domains: confounding, intervention classification, participant selection, deviations from intended interventions, missing data, outcome measurement and result reporting. There was author overlap between papers, with the same groups having published most papers (i.e. Flowers *et al*. [111,113] and Hu *et al*. [103,104, 110, 112]), which might create overlap between study populations. We did not exclude these studies as we considered that the risk of bias would be negligible. Although many studies use similar methods, such as 16S rRNA sequencing, they often lack consistency in reporting key details, making cross-study comparisons difficult. For instance, not all studies specify the primers used, report statistical measures like *P*-values or include alpha and beta diversity metrics.

Most studies acknowledged their limitations due to the lack of control for confounding factors, such as diet and lifestyle (Fig. 2). We also assessed the certainty of evidence using the GRADE approach for any reported correlation between psychotropic use and changes in gut microbial species (Table S2) [114]. Evidence is categorized by high, moderate, low and very low certainty.

### Effects of mood stabilizers on gut microbiome in BP

Our collected research studies yield mixed results. Eight of the studies used sequencing to examine changes in the gut microbiome after psychotropic use (Table 4). There were generally no significant associations between alpha or beta diversity and psychotropic treatment (Table 4). However, in three studies, Lai *et al*. [103] found increased alpha diversity in BP patients after a 4-week treatment of atypical antipsychotics (AAPs), and the Flowers research group noticed a significantly decreased alpha diversity in treated female patients that was not observed in males [111,113]. Three studies found a significant separation between microbiota communities of treated and nontreated patients by beta diversity [103,112, 113].

**Table 4.** Summary of results in studies using sequencing showing a comparison between non-medicated and medicated BP patients

Study	Protocol	Sequencing method	Sequencing result	*α*-/*β*-Diversitytreated vs. untreated	Result
Coello *et al*., 2019 [105]	16S rRNA sequencing	V3–V4 region 16S rRNA2×300 bp (Illumina MiSeq)S-d-Bact-0341-b-S-17S-d-Bact-0785-a-A-21	1,286 OTUs386–428 bp9,225 sequences/sample	Not reported	Psychotropics not correlated with discriminatory *Flavonifractor* presence (*P*>0.05)
Flowers *et al*., 2017 [113]	16S rRNA sequencing	V4 region 16S rRNA2×250 bp (Illumina MiSeq)^†^	Not reported	↓*α* (*P=0.045)‡↓*α* ♀ (*P=0.015)‡*α* ♂ (*P*=0.8)‡*β* (*P=0.04)§	Psychotropics correlated with ↑*Lachnospiraceae* (***P=0.001) and ↓*Akkermansia* (*P=0.03)
Flowers *et al*., 2019 [111]	16S rRNA sequencing	V4 region 16S rRNA2×250 bp (Illumina MiSeq)^†^	2.5k sequences/sample	*α* (*P*=0.12)‡↓*α* ♀ (*P=0.018)‡*α* ♂ (*P*=0.9)‡	Psychotropics correlated with ↓*Alistipes* (*P=0.03)
Hu *et al*., 2019 [112]	16S rRNA sequencing	V3–V4 region 16S rRNA2×250 bp (Illumina MiSeq)341F CCTACGGGNGGCWGCAG785R GACTACHVGGGTATCTAATCC	718 OTUs100–550 bp20k sequences/sample	*α* (*P*>0.05)‡*β* (**P=0.0075)||	Psychotropics correlated with ↑*Proteobacteria*, ↑*Gammaproteobacteria*, ↑*Enterobacteriaceae*, ↑*Enterobacteriales*, ↑*Alphaproteobacteria*, ↑*Paraprevotella*, ↑*Veillonella*, ↑*Lactobacillaceae*, ↑*Lactobacillus*, ↑*Klebsiella*, ↑*Anaeroglobus*, ↑*Collinsella*, ↑*Coriobacteriales*, ↑*Coriobacteriaceae*, ↑*Solobacterium*, ↓*Alistipes*, ↓*Rikenellaceae*
Ioannou *et al*., 2024 [108]	Metagenomic shotgun sequencing	Whole genomeIllumina NovaSeq 6000Reads trimmed Trimmomatic v0.39-2	4 marker genes min.75 bp alignment length min.	*α* (*P*>0.05)¶*β* (*P*>0.05)#	Psychotropics correlated with ↑*Klebsiella* (**P=0.01), ↑*Klebsiella pneumoniae* (**P=0.0002), ↑*Anaeromassilibacillus* (**P=0.005)
Jiang *et al*., 2019 [106]	16S rRNA sequencing	V3–V4 region 16S rRNA(Illumina MiSeq)338F ACTCCTACGGGAGGCAGCAG806R GGACTACHVGGGTWTCTAAT	Not reported	Not reported	Psychotropics correlated with ↑*Enterobacter*, ↓*Ruminococcaceae*, ↓*Faecalibacterium* (*P*=NR)
Lai *et al*., 2012 [103]	Metagenomic sequencing	Whole genomeIllumina NovaSeq 6000	2.258112 TB bases	↑*α* (*P=0.015)¶*β* (*P<0.05)‡‡	Psychotropics correlated with *↑Anaerofustis stercorihominis*, *↑Streptococcus cristatus*, *↑Campylobacter hominis*, *↑Leuconostoc unclassified*,*↑Clostridium bartlettii*,*↑Porphyromonas uenonis*,*↑Anaerococcus vaginalis*, *↑Clostridium perfringens*,*↑Streptococcus parasanguinis*, *↑Clostridium scindens*,*↑Streptococcus salivarius*, *↑Ruminococcus obeum*, *↑Enterobacter cloacae*, *↓Bilophila unclassified*, *↓Enterococcus hirae*, *↓Bifidobacterium dentium* (*P*=NR)
Xi *et al*., 2023 [104]	Metagenomic sequencing	Whole genome	200–400 bp	*α* (*P*>0.05)#*β* (*P*>0.05)‡‡	*↑Eubacterium_biforme*, *↑Weissella_confusa*, *↑Oribacterium_sinus*, *↑TM7_TM7b*, *↑Barnesiella intestinihominis*, *↓Clostridium bartlettii*, *↓Bacteroides sp_2_1_22*, *↓Bacteroides sp_3_1_19* (*P*=NR)

♀, Stratified by sex (female).

♂, stratified by sex (male).

↑, increased diversity/abundance.

↓, decreased diversity/abundance.

*α*, alpha diversity (species variation within a single sample).

*β*, beta diversity (species variation across multiple samples).

min., minimum.

*P≤0.05,

**P≤0.01,

***P≤0.001.

†Primers not reported.

‡Inverse Simpson index.

§Yue and Clayton distance.

||Weighted UniFrac index.

¶Shannon index.

#Robust Aitchison distance.

‡‡Pearson index

k, thousand; NR, not reported; OTU, operational taxonomical unit; *P*, *P*-value.

In a longitudinal monozygotic twin study, microbiome abundance was also at the lowest level following a female patient’s hospitalization and treatments of lamotrigine, lithium and quetiapine [106]. This sex-dependent change is consistent with preclinical studies of AAP-treated rat models like olanzapine and risperidone, where weight gain and microbial composition changes only occurred in females [115,117]. Olanzapine was shown to convert gut microbial composition into an obesogenic profile [89], and risperidone seemed to decrease ‘lean gut microbiota’ [118]. Children and adolescents under chronic risperidone treatment also showed an upregulation of metabolic pathways linked with weight gain [119]. These findings reinforce that AAPs alter the inflammatory milieu and metabolic profile of BP patients drastically, shifting them towards an obesogenic one.

The emergence of antimicrobial resistance among gut bacteria in patients treated with psychotropic medications was a notable concern. The most frequently isolated bacteria from BP patients and HC were *Escherichia coli*, with antibiotic resistance patterns differing between the two groups [107]. Amoxicillin clavulanic acid, cefuroxime, ceftriaxone, cefepime, cefotaxime, meropenem, ciprofloxacin and tetracycline resistance were significantly higher among *Escherichia coli* bacteria isolated from participants taking psychotropic drugs than *Escherichia coli* bacteria isolated from apparently HC. This shows that multidrug-resistant bacteria and extended-spectrum beta-lactamase (ESBL)-producing strains are significantly more prevalent in BP patients taking psychotropics. The overall rate of multidrug resistance among bacteria isolated from patients taking psychotropics was 63%, whereas it accounts for 42.1% among bacteria isolated from HC. Furthermore, the odds of isolating ESBL-producing *Enterobacteriaceae* were notably higher in patients treated with antipsychotic drugs for over a year. This raises questions about the indirect effects of psychotropic medications on microbial ecology, possibly mediated by altered gut environments or medication-induced immune modulation [107].

Gut-brain modules, microbial pathways hypothesized to influence brain function, were not associated with psychiatric symptom severity or medication use [108]. However, quetiapine treatment was linked to 12 microbial modules, 11 of which were enriched in treated BP patients. These included pathways related to leucine biosynthesis, the glyoxylate cycle, haem biosynthesis, complex II succinate/fumarate reductase, the methionine salvage pathway and multiple transport systems (e.g. microcin C, oligogalacturonide, putrescine, arginine, dipeptide and manganese/iron) [112]. Pyruvate ferredoxin oxidoreductase was enriched in non-treated BP [112]. Lai *et al*. and Xi *et al*. did not report any microbial functional changes [103,104].

While some studies reported changes only at the genus level, others also documented changes at the species level. We classified findings of changes in gut microbiome composition into ‘increased abundance’, ‘decreased abundance’ and ‘mixed findings’ (Table 5).

**Table 5.** Changes in gut microbiome composition upon psychotropic treatment

Increased abundance	Decreased abundance	Mixed result
*Alphaproteobacteria* [112]*Anaerococcus vaginalis* [103]*Anaerofustis stercorihominis* [103]*Anaeroglobus* [112]*Anaeromassilibacillus* [108]*Barnesiella intestinihominis* [104]*Campylobacter hominis* [103]*Clostridium perfringens* [103]*Clostridium scindens* [103]*Collinsella* [112]*Coriobacteriaceae* [112]*Coriobacteriales* [112]*Enterobacter* [106]*Enterobacter cloacae* [103]*Enterobacteriales* [112]*Enterobacteriaceae* [112]*Eubacterium biforme* [104]*Eubacterium rectale* [110]*Gammaproteobacteria* [112]*Klebsiella* [108,112]*Klebsiella pneumoniae* [108]*Lachnospiraceae* [113]*Lactobacillaceae* [112]*Lactobacillus* [112]*Leuconostoc unclassified* [103]*Oribacterium sinus* [104]*Paraprevotella* [112]*Porphyromonas uenonis* [103]*Proteobacteria* [112]*Solobacterium* [112]*Streptococcus parasanguinis* [103]*Streptococcus salivarius* [103]*Streptococcus cristatus* [103]*Veillonella* [112]*Weissella confusa* [104]	*Akkermansia* [113]*Alistipes* [111,112]*Bacteroides_sp_2_1_22* [104]*Bacteroides_sp_3_1_19* [104]*Bilophila unclassified* [103]*Enterococcus hirae* [103]*Faecalibacterium* [106]*Rikenellaceae* [112]*Sutterella* [113]	*Bifidobacteria* [110]*Bifidobacterium dentium* [103]*Clostridium bartlettii* [103,104]*Ruminococcaceae* [106]*Ruminococcus obeum* [103]

### Increased bacteria abundance post-treatment

Increased abundance of 23 genera and 18 bacterial species was observed in BP patients treated with antipsychotics and lithium, and anticonvulsant and antidepressant uses were not associated with any of the taxa (Table 5). These specific species include *Anaerococcus vaginalis*, *Anaerofustis stercorihominis*, *Barnesiella intestinihominis*, *Campylobacter hominis*, *Clostridium perfringens*, *Clostridium scindens*, *Enterobacter cloacae*, *Eubacterium biforme*, *Eubacterium rectale*, *Klebsiella pneumoniae*, *Leuconostoc unclassified*, *Oribacterium sinus*, *Porphyromonas uenonis*, *Ruminococcus obeum*, *Streptococcus parasanguinis*, *Streptococcus salivarius*, *Streptococcus cristatus* and *Weissella confusa*. These encompass a diverse range of species, including both Gram-positive and Gram-negative bacteria. Many Gram-positive species, such as *Lactobacillus*, are well-known for their roles in human microbiota, especially in the gut, and are involved in processes like fermentation, digestion and the maintenance of gut health [120,121]. *Eubacterium biforme* and *Eubacterium rectale* produce SCFAs like butyrate, maintaining gut barrier integrity and reducing inflammation [122]. Their increased abundance could indicate adaptive microbial changes or therapeutic effects aimed at restoring gut homeostasis in treated patients. *Clostridium Cluster IV* is a butyrate-producing bacterium [123] and belongs to beneficial bacteria [110]. However, some of these bacteria are pathogenic and can cause severe infection. *Clostridium perfringens* is an enteric pathogen that can produce various toxins and hydrolytic enzymes associated with intestinal diseases in humans and animals [103,124]. Gram-negative bacteria like *Campylobacter hominis* and *K. pneumoniae* are also notable for their clinical significance; *K. pneumoniae* are often opportunistic pathogens implicated in the urinary tract [125,126], while *Campylobacter hominis* is a known pathogen in gastrointestinal diseases [127,128].

In a longitudinal monozygotic twin study, KEGG pathway level 3 LPS biosynthesis genes were over-represented in the gut following hospitalization and treatments of lamotrigine, lithium and quetiapine (active state) [106]. LPSs are bacterial surface glycolipids produced by Gram-negative bacteria that can stimulate inflammation in the gut microbiome [129]. During the remissive state, LPS biosynthesis genes are underrepresented compared with the active stage of BP, leading the patient’s gut microbiome to become more similar to that of their monozygotic healthy twin. Throughout the responsive state (discharge and lithium withdrawal) and remissive (outpatient visit) BP state, *Ruminococcaceae* and *Faecalibacterium* abundance also increased significantly while *Enterobacter* declined [106]. This is consistent with previous studies on the general effects of lithium on the gut microbiome [90,130]. These results suggest that gut microbial function partially recovered after patients achieved full remission. Moreover, the overrepresented LPS biosynthesis genes may be related to the overgrowth of *Enterobacter* during the active stage, and patients’ gut microbiome compositions were corrected as they moved towards a healthy status [106].

### Decreased bacteria abundance post-treatment

Following a 4-week quetiapine treatment, the abundance of *Akkermansia* [113], *Alistipes* [111,112], *Bacteroides_sp_2_1_22* [104], *Bacteroides_sp_3_1_19* [104], *Bilophila unclassified* [103], *Enterococcus hirae* [103], *Faecalibacterium* [106], *Ruminococcaceae* [106] and *Sutterella* [113]. *Alistipes* bacteria are within the *Bacteroidetes* phylum, enriched in untreated BP patients [112] and inversely associated with obesity and an animal-rich diet [131,132]. *Akkermansia* was significantly decreased in non-obese patients treated with AAPs [113]. This genus was previously inversely correlated with inflammation, insulin resistance and lipid metabolism [133]. Nevertheless, in two studies, no significant difference was found in bacterial counts before and after treatment [105,134].

### Treatment response classification

In three studies, based on the degree of improvement in depression severity post-treatment (quetiapine) compared with baseline (HAMD-17/HDRS-124/HDRS-17 change ≥50%), patients were classified into responder and non-responder groups, with the responders’ scores significantly lower than non-responders [103,104, 112] (Table 6). These studies highlighted that the comparison between responders and non-responders is separate from the treated vs. non-treated distinction.

**Table 6.** Species enriched in 4-week quetiapine responders and non-responders

Study	Treatment response criterion	Responders’ enriched species	Non-responders’ enriched species
Hu *et al*., 2019 [112]	HDRS-17 score reduction from baseline of at least 50% after a 4-week quetiapine treatment	*Paraprevotella* (*P*=0.0072)*Lachnospira* (*P*=0.0356)*TM7 genera incertae sedis* (*P*=0.0366)	*Acinetobacter* (*P*=0.0151)*Asaccharobacter* (*P*=0.0152)*Eubacterium* (*P*=0.0023)*Lactococcus* (*P*=0.01523)*Lactobacillus* (*P*=0.0362)*Achromobacter* (*P*=0.0151)*Bifidobacterium* (*P*=0.0356)
Lai *et al*., 2012 [103]	HDRS-124 score reduction from baseline of at least 50% after a 4-week quetiapine treatment	*Streptococcus sanguinisSubdoligranulum sp_4_3_54A2FAAActinomyces orisGordonibacter pamelaeaeBlautia productaBacteroides clarusParaprevotella unclassifiedEubacterium biformeWeissella confusaRuminococcus torquesClostridium sp_ATCC_BAA_442Gemella unclassifiedEnterococcus faeciumCollinsella unclassifiedParaprevotella xylaniphilaDorea formicigeneransEubacterium limosumEubacterium ramulus* (*P*=NR)	*Bacteroides xylanisolvensClostridium difficileBifidobacterium bifidumLactobacillus crispatus* (*P*=NR)
Xi *et al*., 2023 [104]	HAMD-17 score reduction from baseline of at least 50% after a 4-week quetiapine treatment	*Eubacterium biformeWeissella confusaOribacterium sinusTM7_TM7b Barnesiella_intestinihominis* (*P*=NR)	*Clostridium bartlettiiBacteroides_sp_2_1_22Bacteroides_sp_3_1_19* (*P*=NR)

HAMD, Hamilton Depression Rating Scale; HDRS, Hamilton Depression Rating Scale; NR, not reported; P, P-value.

Several genera and species were consistently enriched in quetiapine responders, particularly members of the *Paraprevotella* genus, which were identified by both Lai *et al*. and Hu *et al*. [103,112]. In Lai *et al*. and Xi *et al*., a broader range of responder-associated taxa was noted, including beneficial commensals such as *Eubacterium biforme*, *Weissella confusa,* and *Subdoligranulum* sp*.* Notably, *Eubacterium biforme* and *Weissella confusa* were enriched in responders in both Lai *et al*. and Xi *et al*.’s studies, suggesting potential cross-study reproducibility [103,104]. These microbes may contribute to a more favourable gut environment or immune modulation that enhances treatment response.

Conversely, non-responders displayed an enrichment of species often associated with inflammation or opportunistic pathogenicity. For instance, Hu *et al*. and Lai *et al*. both reported an increase in *Bifidobacterium* and *Lactobacillus* species in non-responders, which contrasts with their traditional probiotic role and raises questions about strain-level differences or context-specific effects in psychiatric populations [103,112, 120, 135,138]. Additionally, non-responders in Lai *et al*. showed enrichment of *Clostridium difficile* and *Bacteroides xylanisolvens*, species that may indicate microbial dysbiosis or inflammatory potential [103,139, 140]. Xi *et al*. also found enrichment of *Clostridium bartlettii* and certain *Bacteroides* species in non-responders [104].

### Mixed findings

The abundance of *Clostridium bartlettii* varied across two studies [103,104] (Table 5). Both studies classified the BP patients as quetiapine responders and non-responders after a 4-week treatment based on HAMD changes. Lai *et al*. identified an increased abundance of *Clostridium bartlettii* following quetiapine treatment in the study. This was accompanied by improvement in depressive symptoms following treatment, which appears to correlate with crucial emotion and behaviour recovery in BP patients but increased metabolic dysfunction [103]. Conversely, Xi *et al*., from the same research group, observed elevated *Clostridium bartlettii* levels in quetiapine non-responders, linking it to 5-HT deficiency and poorer remission outcomes [104] (Table 6). They argued that as that species has been linked to 5-HT deficiency [141], patients with excess *Clostridium bartlettii* may not do well in remission post-quetiapine treatment [104]. They also added that *Clostridium bartlettii* had been associated with increased weight gain [103,142], with a decreased faecal abundance of *Clostridium bartlettii* following metformin treatment in three trials [143,145]. These findings suggest that the species may have a complex role in quetiapine treatment responses, potentially influencing metabolic dysfunction, weight gain and mood regulation. However, whether and how the species has detrimental or rescuing effects on BP symptoms is unclear. Psychotropics (particularly quetiapine) may partially improve mood symptoms through interactions with beneficial bacteria while increasing the risk of metabolic disturbance in more susceptible patients through interactions with other species of gut bacteria.

The findings for *Bifidobacteria* were also inconsistent. One study reported a significant increase in *Bifidobacteria* counts [110], whereas another found a decrease in *Bifidobacterium dentium* after quetiapine treatment [103] (Table 5). In a study conducted by Lu *et al*., after a 4-week quetiapine treatment, the populations of both anaerobic *Bifidobacteria* and *Eubacterium rectale* proliferated compared with baseline, coinciding with a decline in the MADRS score and an increase in *Bifidobacteria-to-Enterobacteriaceae* (B/E) ratio [110]. The authors suggested that these bacteria could serve as biomarkers for therapeutic effects, but their lack of a placebo control leaves causality unclear (Fig. 2). Interestingly, in a separate study conducted by the same group, a 4-week quetiapine treatment decreased the abundance of *Bifidobacterium dentium*, implying its potential role in improved depressive symptoms post-treatment [103]. Nevertheless, the absence of HCs in their fMRI analysis presents some biases and limits these associations (Fig. 2).

The findings on the abundance of *Ruminococcus obeum* species and the family *Ruminococcaceae* following 4 weeks of quetiapine treatment present another conflict. While one study shows an increase in *Ruminococcus obeum* following quetiapine treatment [97], another identifies an ongoing increase in *Ruminococcaceae* during the responsive and remissive states of BP, despite initial decreases in the active state [106]. This is particularly intriguing given that this same genus was enriched in the HC group. This warrants further investigation into the mechanistic pathways of the bacteria and their metabolites in BD progression and therapy.

### Correlated functional imaging changes

**Fig. 3.**
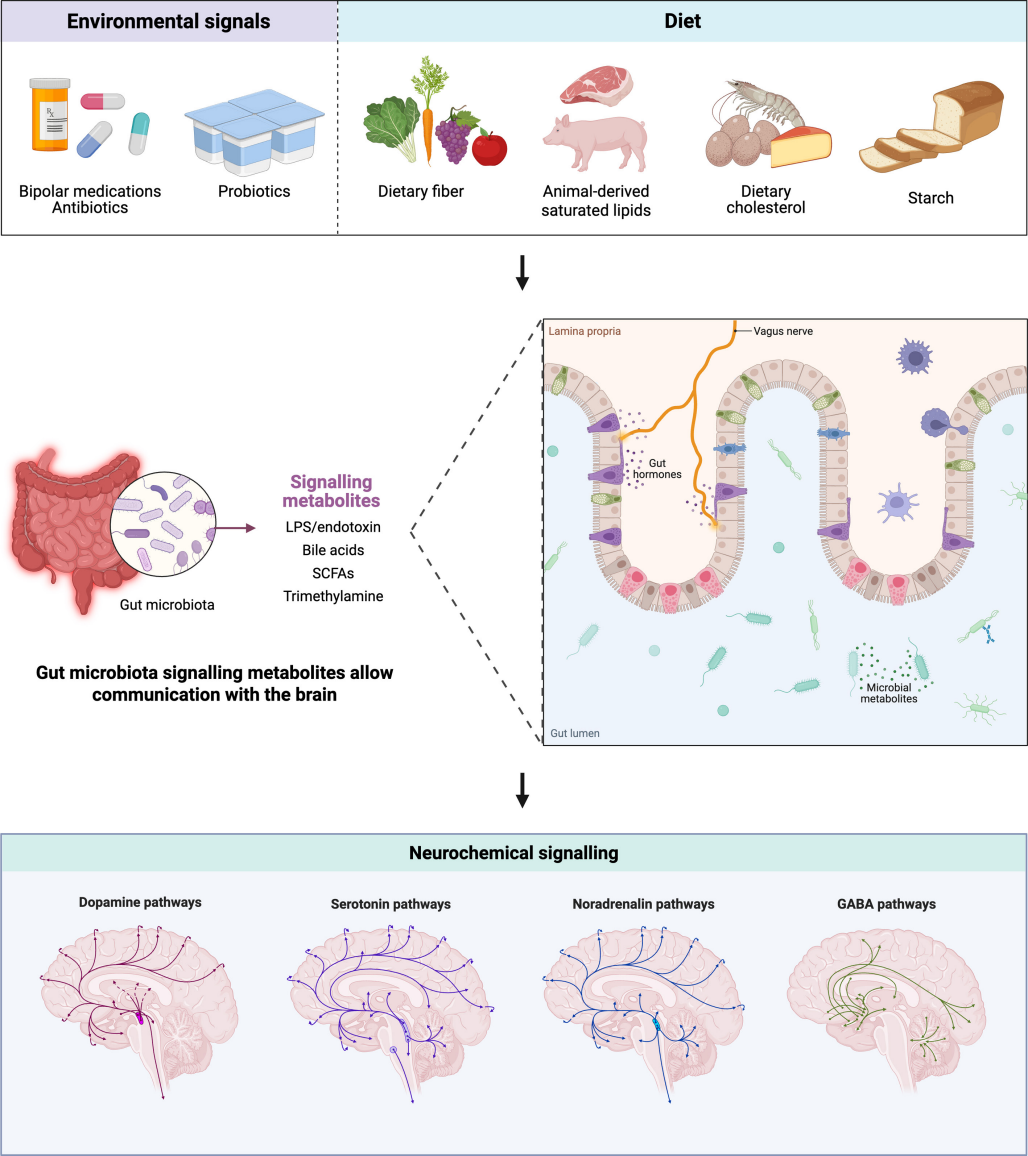
Proposed effects of environmental factors on the gut microbiome composition and neurochemical signalling in BP.

Two studies from our search employed fMRI to investigate the correlation between gut bacterial species and neural activity [103,104]. However, Lai *et al*. only collected fMRI results from untreated BP patients [103]. In contrast, Xi *et al*. collected fMRI data from HC and all BP patients before and after they underwent a 4-week quetiapine treatment [104]. They showed that the FC and grey matter volume of quetiapine-responding BP patients were closer to those of HC than non-responders, suggesting that successful quetiapine treatment may restore some neural function and connectivity to a more normative state. Moreover, quetiapine-responding patients had lower intra-network FC within DMN and between the DMN and other networks (i.e. frontoparietal network and ventral attention). This lower FC was negatively correlated with *TM7_TM7b* (bacteria from the *Saccharibacteria* phylum), *Weissella confusa*, and positively correlated with *Clostridium bartlettii*. FC within the DMN is positively correlated with 5-HT signal transduction [146], which is related to the primary action of quetiapine (e.g. blocking dopamine D2 [147] and 5-HT_2A_ receptors [148,149], while activating 5-HT_1A_ [150]). Significantly higher DMN connections indicate that non-responders have dysfunctional or abnormal 5-HT signal transduction, making 5-HT more difficult for the quetiapine to bind to the 5-HT receptor [104] (Fig. 3).

These imaging findings suggest that quetiapine non-responders may have additionally abnormal 5-HT signalling, leading to insufficient quetiapine binding at key receptors. This dysfunction within the 5-HT system contributes to the persistent DMN hyperconnectivity observed, amplifying an already maladaptive neural state.

## Discussion

The gut-brain axis is a bidirectional communication system involving the CNS, enteric nervous system and gut microbiota. The gut microbiome interacts with the brain via neurotransmitters, metabolites and hormones [151]. Thus, changes to the CNS in psychiatric disorders, such as neurotransmitter dysregulations, can affect the gastrointestinal microenvironment and vice versa [152]. Studying the effects of medications on the gut microbiome in BP is essential for advancing our understanding of treatment mechanisms and improving patient outcomes. A significant number of individuals with BP do not respond adequately to existing medications such as mood stabilizers, antipsychotics or antidepressants [153,154]. Gut dysbiosis may perpetuate systemic inflammation or disrupt neurochemical signalling, such as serotonin and GABA pathways, contributing to poor treatment outcomes. By examining how medications influence gut bacteria, researchers can uncover underlying mechanisms of treatment resistance and identify new personalized strategies to support patients who do not respond to conventional therapies. The gut microbiome is highly individualized and shaped by diet, lifestyle, genetics and environment [155]. Medications may impact each person’s microbiome differently, contributing to variability in therapeutic outcomes. By studying these effects, researchers can develop microbiome-based biomarkers to predict how a patient will respond to specific treatments, enabling clinicians to tailor therapies to individual needs. This approach aligns with the growing emphasis on precision medicine in mental health care [156].

This review provides the most comprehensive analysis to date on the effects of BP treatments on the gut microbiome, with comparisons between HCs and untreated and treated BP. We found that quetiapine and lithium were associated with an increased abundance of various gut bacteria species. Specifically, elevated levels of *Clostridium perfringens* and *Enterobacter cloacae –* opportunistic pathogens *–* suggest gut dysbiosis following treatment [103]. On the other hand, the increased abundance *of Eubacterium biforme*, *Eubacterium rectale* and *Ruminococcus obeum*, which produce beneficial SCFAs, indicates that quetiapine may have some positive effects on restoring gut homeostasis in treated patients [103,104, 110]. Additionally, the use of psychotropic drugs may contribute to antibiotic resistance, as observed in studies of *Escherichia coli* from patients on these medications [107]. This suggests that psychotropic drugs could promote the emergence of multidrug-resistant bacteria. Some studies also propose that gut microbiome composition could serve as a non-invasive biomarker for diagnosing and predicting treatment outcomes in mental health disorders like BP.

### Study limitations

This review presents some limitations. We restricted the scope of this review to studies published in peer-reviewed journals, which introduces a potential for publication bias. Studies reporting significant or positive findings are more likely to be published, while those presenting null or negative results may remain unpublished. This bias could distort the conclusions regarding the effects of medications on the gut microbiome. Incorporating grey literature, such as conference proceedings or preprints, could mitigate this limitation by providing a more comprehensive and balanced representation of the available evidence. Such sources often include novel or preliminary findings, which are particularly valuable in rapidly evolving fields like microbiome research. Moreover, this review did not conduct any statistical analyses due to the substantial heterogeneity in the methodologies of the studies included. Variations in study design, populations, analytical techniques and reporting standards precluded meaningful quantitative synthesis, further limiting the robustness of the conclusions.

Potential sources of bias in these studies encompass variations in sample characteristics, notably the marked clinical heterogeneity within the BP cohort, geographical and dietary influences and potential author overlap between papers (i.e. Flowers *et al*., – same groups have published most papers). As a result, it is challenging to interpret the disparities in measures of diversity and abundance between studies. Only four studies were interventional, where participants underwent a 3–4-week treatment with psychotropic medications [103,104, 110, 111]. These studies employed a fixed dose of quetiapine (300 mg day^−1^) without a discernible dose-response correlation. The remaining eight studies were observational, where researchers monitored and compared the effects of psychotropic medications between individuals who were taking psychotropics and those who did not. There was significant heterogeneity between studies. Out of the total number of studies conducted (12 in total), only a subset shared comparability due to their utilization of a cross-sectional cohort design for comparing individuals with BP to HC groups. These studies generally featured modest sample sizes; in some studies, the patient group did not receive placebo treatment as a control [110].

Geographical variation may present another confounder since regional diets can vary greatly. While antibiotics, probiotics and prebiotics were prohibited in many studies, it is essential to note that participants did not adhere to a standardized diet or physical activity, both pivotal environmental factors influencing gut microbiota composition [157]. Some studies also allowed for a combination of medications in addition to quetiapine treatment, so underlying polypharmacy precludes us from drawing definitive conclusions. It would be difficult to control for this in future studies since most BP patients exist on an affective mood spectrum, which necessitates the use of multiple medications [158]. Additionally, participant age was not matched between the patient and control groups, and gut microbiome composition changes rapidly with age [159,160].

Only a subset of included studies was comparable; however, they often had small sample sizes, and in some cases, the patient group did not receive a placebo treatment as a control [110]. None of the studies examined differences in AAP responses between BP-I and BP-II. Nevertheless, the findings may provide important preliminary information, as the study began at the time of diagnosis and continued with follow-up assessments throughout the treatment period. Some studies examined gut microbiota within the context of various psychiatric conditions, such as SCZ, alongside BP and explored diverse treatments, including starch and probiotics; however, the outcomes of these investigations fell beyond the scope of this review.

Another constraint pertained to the dropout rate among patients who initially received treatments, leading to the re-evaluation and final inclusion of only a subset of the patient population [112]. Consequently, certain studies could not elucidate whether alterations in microbiota traits were tied to treatments. To establish a cause-and-effect relationship between BP gut microbiota and bipolar pharmacological treatments, a longitudinal cross-sectional cohort design is imperative. It is worth mentioning that the distinction between BP-I and BP-II in relation to gut microbiome composition changes following treatment was not examined in the studies.

Many of the included papers recruited a broad spectrum of psychiatric patients, not exclusively individuals with BP. Many mood and psychiatric disorders, including BP, SCZ and MDD, are increasingly recognized to exist along a continuum rather than as distinct, isolated conditions. These disorders often share overlapping symptoms – such as mood instability, cognitive impairments and psychotic features – as well as common biological underpinnings, including genetic risk factors and neurochemical imbalances [161,165]. This diagnostic overlap poses significant challenges for clinical research and participant recruitment, making it difficult to identify and enrol participants who fit neatly into a single diagnostic category [166,167]. Consequently, studies that aim to focus solely on BP often face issues related to diagnostic heterogeneity, which can limit the specificity and generalizability of their findings. Our goal was to include all relevant studies that met our inclusion criteria; thus, our search returned studies that also recruited patients with SCZ, MDD and other psychiatric conditions. We deliberately included these studies for transparency and to avoid excluding potentially important data on medication-induced microbiome changes. However, this approach means that the reported gut microbiome alterations may not be entirely specific to BP. While future studies that isolate BP populations may be challenging to conduct, they would be valuable for further clarifying the gut microbiome’s role in this population.

There is an established risk that antidepressants may induce a switch to mania or contribute to the development of rapid cycling in individuals with BP [168,170]. One study in our data included a BP patient who underwent all active, responsive and remissive phases [106]. Given that antidepressants were included as part of the psychotropic medications in many of the studies reviewed, this raises the concern that some of the participants diagnosed with BP could have experienced such a switch or worsening of their condition. This is particularly relevant since the effects of psychotropic medications on the gut microbiome may differ between individuals with MDD and those with BP, further complicating the interpretation of the findings. As a result, we cannot fully isolate the impact of psychotropic medications on the gut microbiome in individuals with BP specifically. This limitation is particularly important when considering the generalizability of the findings and the need for further studies focusing specifically on bipolar patients who are not concurrently treated with antidepressants.

The majority of included studies focus on the effects of specific medications like quetiapine and lithium, potentially neglecting other treatments used in BP, such as valproate. This limited scope might hinder the generalizability of findings to all BP medications. Studies assessing the gut microbiome and BP treatments likely vary in methodologies, including participant demographics, dosage, treatment duration and microbiome assessment techniques. This heterogeneity complicates direct comparisons and synthesis of results. Additionally, distinguishing the effects of psychotropic medications from the underlying disease itself remains a significant challenge. Many BP patients are on multiple medications, and factors like disease severity or duration may interact with the effects of psychotropic drugs, making it difficult to isolate the precise impact of any one treatment on the microbiome.

Many studies on the gut microbiome are cross-sectional, providing a snapshot of microbiome composition at a single time point. These studies may fail to capture the dynamic changes in gut bacteria over the course of treatment, limiting insights into causality. Many microbiome studies involve small cohorts, limiting statistical power and the ability to detect subtle or clinically meaningful changes in the gut microbiome. While the studies discuss changes in microbiome composition, they lack mechanistic insights and may not provide sufficient mechanistic understanding of how these changes interact with psychiatric treatments and contribute to treatment resistance or efficacy. Although we highlight immediate changes in gut bacteria associated with treatments (i.e. 4 weeks), the studies did not sufficiently address long-term impacts, such as the potential development of antibiotic resistance or chronic dysbiosis.

Differences in biological sampling processes (e.g. 16S rRNA sequencing, shotgun metagenomics, ELISA and PCR) and analytical pipelines could introduce variability in results, making it difficult to draw consistent conclusions. 16S rRNA sequencing, for example, typically targets a specific region of the bacterial genome, which may provide a less comprehensive view of the microbial community compared with shotgun metagenomics, which offers a higher resolution by sequencing the entire genetic content of the microbiome. Therefore, the choice of sequencing method can affect the depth of microbial diversity captured, with 16S sequencing potentially missing certain species or functional information that shotgun metagenomics might detect. Additionally, the specific regions targeted in 16S rRNA sequencing (e.g. the V3–V4 region) vary between studies, potentially leading to differences in the types of bacteria identified and their relative abundances. These methodological differences, coupled with variations in data processing pipelines and bioinformatic approaches, can contribute to heterogeneity across studies and influence the overall conclusions drawn regarding the relationship between psychotropic medications and the gut microbiome in BP. Given these variations, caution must be exercised when comparing studies, and future research should aim for standardized methods to enhance the reliability and comparability of findings.

Furthermore, although several studies employ similar methodologies, such as 16S rRNA sequencing, they often do not report consistent methodological or analytical details, making it difficult to compare findings across studies. For example, while 16S rRNA sequencing is commonly used to assess gut microbiota composition, not all studies specify the primers used. This can significantly influence the regions of microbial DNA amplified and the taxa detected. Additionally, there is variability in reporting statistical details, such as *P*-values or effect sizes, and in the presentation of diversity metrics like alpha and beta diversity (Table 6). This inconsistency hampers the ability to synthesize results meaningfully and limits the reproducibility and comparability of microbiome research across different cohorts.

Overall, the findings across these studies suggest that gut microbiota composition may be predictive of quetiapine treatment response in depression. Responder profiles appear to favour SCFA-producing and potentially anti-inflammatory bacteria, while non-responders exhibit a microbiota composition that may reflect a dysbiotic or pro-inflammatory state. However, discrepancies across studies and the lack of statistics for many species warrant caution. Further research is needed to validate these microbial signatures and determine causality or potential therapeutic modulation via microbiome-targeted interventions.

### Preclinical findings

Animal models are widely used to study the effects of medications on the gut microbiome. Preclinical studies in animal models have increasingly highlighted psychotropic medications’ significant and diverse effects on the gut microbiome, providing mechanistic insights that complement clinical observations [171].

#### Antidepressants

In rodent models, administration of antidepressants escitalopram and fluoxetine (SSRIs), as well as venlafaxine (serotonin–norepinephrine reuptake inhibitor), significantly increased epithelial permeability in the distal ileum compared with vehicle-treated controls, with no notable changes observed in the distal colon [90]. These same antidepressants, along with duloxetine, were also associated with reduced microbial richness and increased beta diversity in the gut microbiota [172]. Since higher microbial diversity is generally associated with better health outcomes, and reduced richness has been linked to conditions such as inflammatory bowel disease [173,174] and obesity [175], these findings raise concerns about gastrointestinal side effects of antidepressant treatment.

However, evidence on the impact of antidepressants on gut microbiota remains inconsistent. For example, another study reported that fluoxetine and amitriptyline increased alpha diversity in depression-induced rats [176]. Fluoxetine, in particular, has been associated with gut dysbiosis, though the nature of this shift varies across studies. One report found that fluoxetine increased the *Firmicutes*/*Bacteroidetes* ratio [177], while another observed a decrease in *Firmicutes* and an increase in *Bacteroidetes* following treatment with both fluoxetine and amitriptyline [176]. These discrepancies may be attributed to species-specific differences between mice and rats.

Functionally, fluoxetine and amitriptyline were shown to reduce microbial pathways related to membrane transport, carbohydrate metabolism and signal transduction [176]. Taxonomically, fluoxetine increased the relative abundance of several genera, including *Alistipes*, *Lachnospiraceae*, *Coriobacteriaceae*, *Anaerotruncus*, *Ruminiclostridium_5* and *Lachnoclostridium* [177]. Notably, *Alistipes* species have been implicated in colitis and other gastrointestinal disturbances [178,179]. As indole-positive bacteria, *Alistipes* may alter tryptophan availability – an essential precursor to 5-HT – potentially disrupting the intestinal serotonergic system [180,181].

Additionally, both amitriptyline and fluoxetine significantly increased the abundance of *Bacteroides*, *Parabacteroides*, *Butyricimonas*, *Acetatifactor*, *Porphyromonadaceae* and *Tyzzerella* [177]. Some members of *Porphyromonadaceae* have been linked to neuroinflammation [182,183], while *Bacteroides* and *Parabacteroides* are known to express pathways involved in GABA synthesis – an inhibitory neurotransmitter critical in stress regulation [184]. Given that antidepressants ultimately enhance GABAergic signalling [185], these microbial changes may reflect or contribute to their pharmacological effects. *Butyricimonas*, a known butyrate producer, has anti-inflammatory properties and supports gut health [186,187].

Fluoxetine also decreased the abundance of *Prevotella 7*, *Prevotella 9* and *Succinivibrio* [90,174]. Other antidepressants – including escitalopram, venlafaxine, duloxetine and desipramine – reduced levels of *Ruminococcus* and *Adlercreutzia*, with several selectively depleting *Adlercreutzia equolifaciens* [172]. Interestingly, amitriptyline increased *Butyricimonas* more than fluoxetine [181], suggesting drug-specific microbial effects.

Collectively, these findings highlight the complex and often contradictory effects of antidepressants on the gut microbiome. Each drug appears to exert distinct, and sometimes opposing, influences on microbial diversity, composition and function, underscoring the need for further research to fully elucidate their impact on gut-brain axis interactions.

#### Antipsychotics

The impact of psychotropic agents on the gut microbiota is highly variable and agent specific. Notably, lithium, valproate and aripiprazole treatment in animal models significantly increased microbial alpha diversity, indicating enhanced microbial richness. These agents also induced distinct changes in the abundance of specific bacterial taxa and SCFA profiles. For instance, valproate administration decreased propionate and butyrate levels while increasing isovalerate, whereas aripiprazole led to elevated acetate and isovalerate levels. Other SCFAs, such as valerate and isobutyrate, remained unaffected by any treatment [90].

At the taxonomic level, lithium, valproate and aripiprazole increased the relative abundance of several bacterial genera, including *Clostridium sensu stricto 1*, *Ruminiclostridium 5*, *Intestinibacter*, *Eubacterium coprostanoligens*, *Peptoclostridium*, *Eubacterium oxidoreducens*, *Christensenellaceae uncultured* and members of *Clostridia Family XIII* [90]. The increase in abundance of *Clostridium* and *Eubacterium* seems to be consistent with our findings (Table 5).

At the phylum level, lithium and valproate increased *Actinobacteria*, while both also reduced *Bacteroidetes*. Aripiprazole increased *Firmicutes*, and fluoxetine decreased *Deferribacteres*. At the family level, lithium, valproate and aripiprazole significantly elevated *Peptostreptococcaceae*, *Clostridiaceae* and *Ruminococcaceae*. At the genus level, lithium increased uncultured *Ruminococcaceae* and reduced *Bacteroides* and *Ruminococcus 1*; valproate similarly increased uncultured *Ruminococcaceae* while decreasing *S24-7*; aripiprazole decreased *Ruminococcus 1*. None of the treatments had a notable effect on *Lachnospiraceae* (uncultured), *Akkermansia* or *Oscillibacter* [90].

Aripiprazole was also associated with increased ileal permeability, though this effect is likely unrelated to SERT activity and may involve other 5-HT receptor pathways. Importantly, this increase in permeability was region specific, with minimal changes observed in the colon. Future studies are needed to elucidate whether microbiota shifts under aripiprazole are causally linked to these permeability changes [90].

Antipsychotics, particularly second-generation agents like olanzapine and risperidone, have been shown to profoundly alter gut microbial communities. Chronic olanzapine exposure in rodents led to reduced microbial diversity, a consistent increase in *Firmicutes* and a decrease in *Bacteroidetes –* a microbial profile often associated with metabolic dysfunction, including weight gain and insulin resistance [88]. Specifically, olanzapine increased the abundance of *Erysipelotrichia* (phylum *Firmicutes*) and *Gammaproteobacteria* (phylum *Proteobacteria*), while decreasing *Bacteroidia* (phylum *Bacteroidetes*) [89]. Notably, this microbial shift was linked to weight gain. Germ-free mice did not exhibit the same weight gain until colonized with olanzapine-associated microbiota, suggesting a causal role of the microbiome [90].

Similarly, risperidone treatment altered the microbial landscape by increasing the relative abundance of *Firmicutes* (notably *Allobaculum* and *Turicibacter*, members of the *Erysipelotrichaceae*) and decreasing *Bacteroidetes*. Within *Firmicutes*, *Lactobacillus* was more abundant in control animals, whereas *Allobaculum* dominated in treated animals. Within *Bacteroidetes*, *Alistipes* was depleted while *Bacteroides* was enriched [87,118]. Significant enrichment in *Allobaculum* and *Turicibacter* spp., both members of the *Firmicutes* family *Erysipelotrichaceae. Anaeroplasma* spp., a member of the *Mollicutes* class, was also found to be more abundant following risperidone treatment [87]. There was a depletion of *Alistipes* spp. and *Akkermansia* spp. in risperidone-treated animals compared with controls, both of which have been associated with a lean microbiota [87,118]. Additionally, risperidone-treated mice exhibited reduced energy expenditure and greater weight gain compared with controls, despite similar food intake. These metabolic effects were transferable through faecal microbiota transplantation (FMT), implicating the gut microbiome in the underlying mechanism [90].

Interestingly, sex-specific effects were observed with olanzapine; female rats showed increased weight gain, inflammatory markers and metabolic disturbances not seen in males, highlighting sex as a potential modulator of psychotropic-induced microbiome changes [115]. Other host influencing variables include drug dose, treatment duration and route of administration [188].

#### Ketamine

Ketamine has emerged as a promising therapeutic option, demonstrating rapid and robust symptom relief in adults with treatment-resistant mood disorders [189,190]. In particular, intravenous ketamine has shown strong short-term efficacy in reducing suicidality, including suicidal ideation [191], and appears highly effective for patients with treatment-resistant BP [192]. The antidepressant effects of low-dose ketamine are well supported by both preclinical and clinical evidence [101,157, 193,196].

Beyond its CNS effects, ketamine significantly alters gut microbiome composition. Chronic administration of low-dose ketamine increases the abundance of *Lactobacillus*, *Sarcina* and *Turicibacter*, while reducing *Mucispirillum* and *Ruminococcus*. It also selectively decreases the phyla *Deferribacteres* and *Tenericutes* compared with saline controls. Notably, elevated levels of *Mucispirillum* and *Ruminococcus* have been associated with gut inflammation and neuropsychiatric symptoms [197,199]. *Mucispirillum*, a Gram-negative anaerobe, constantly produces LPS, a known inflammatory trigger [200,201]. *Ruminococcus* species have been linked to irritable bowel syndrome, intestinal inflammation and stress-induced depressive behaviour [200,202,205]. Thus, ketamine’s reduction of these pro-inflammatory taxa may contribute to its antidepressant and anti-inflammatory effects [206].

Conversely, increased levels of *Lactobacillus*, *Turicibacter* and *Sarcina* taxa, often found in lower abundance in individuals with depression and other inflammatory conditions [207,209], may support a more balanced and resilient gut ecosystem. *Sarcina* in particular has been implicated in modulating inflammation [210], suggesting that its enrichment could also underlie some of ketamine’s therapeutic effects. At the family level, ketamine significantly increased the abundance of *Tuberibacteraceae*, *Clostridiaceae* and *Lactobacillaceae*, while decreasing *Deferribacteraceae* and *Ruminococcaceae*. These shifts further suggest that ketamine may promote gut microbial homeostasis, which could, in turn, influence systemic inflammation and mood regulation [211].

Despite these promising findings, significant challenges remain in translating preclinical gut microbiome data to human contexts. The composition and functionality of gut microbiota in laboratory animals differ markedly from those in humans. Key microbial phyla may be underrepresented or absent in animal models, limiting the generalizability of results [212]. Moreover, species-specific differences in drug metabolism affect the concentration of ketamine and its metabolites that reach the gut, potentially altering microbiome interactions [213]. Controlled housing, uniform diets and sterile conditions in laboratory settings, while beneficial for reducing experimental variability, fail to replicate the complexity of human environmental exposures and lifestyle factors.

Common practices such as the use of antibiotic-treated or germ-free animals re-colonized with human microbiota only partially replicate the diverse and stable microbial ecosystems found in human guts [214,215]. These models may result in selective colonization or omit key microbial species that are crucial for accurate drug-microbiome interaction studies. Furthermore, differences in immune function and behaviour between species complicate the extrapolation of microbiome-driven outcomes such as immune activation or neurobehavioural responses. These limitations highlight the need for more sophisticated models and caution in interpreting preclinical findings when considering ketamine’s effects on the human gut-brain axis.

### Gaps in current clinical research

Human studies offer more clinically relevant insights into drug-microbiome interactions but come with their own set of limitations. One major issue is the high degree of interindividual variability in the human gut microbiome. Factors such as genetics, diet, age, geography, medication history and underlying health conditions all influence microbiome composition, making it difficult to identify consistent drug-induced effects across diverse populations. Compounding this problem are numerous confounding variables in observational studies – such as polypharmacy, dietary habits and comorbid conditions – that make it hard to isolate the specific impact of a medication.

Ethical and logistical constraints further limit experimental designs in human research. Researchers cannot ethically administer potentially harmful interventions or dramatically alter a person’s microbiome. Longitudinal studies, while valuable, require frequent sampling and long-term follow-up, which can be expensive and difficult to maintain. Even when data are collected, most studies rely on faecal samples, which provide only a snapshot of microbial communities and often miss subtle or localized changes, such as those occurring at the mucosal surface.

Another important limitation is the complexity of medication regimens in patients with mood disorders. Most individuals with mood disorders, including BP, are typically prescribed a combination of medications, which may include antidepressants, mood stabilizers, antipsychotics and other psychotropic drugs. These medications often have independent and diverse effects on the gut microbiome, complicating the interpretation of findings. The heterogeneous nature of these treatment protocols makes it challenging to isolate the specific impact of any single drug or class of drugs on the gut microbiota, as the drugs themselves could be altering the microbiota in ways that confound the relationship between BP and the gut microbiome. Additionally, given the nature of psychiatric disorders, where symptom management often requires a mixed approach with multiple medications, the influence of each drug on the microbiome may be additive or interactive, further confounding the results. Consequently, the conclusions that extend beyond the data synthesis risk overestimating the direct impact of psychotropic drugs on the microbiome, without accounting for the confounding effects of prior and concurrent medication use.

While human studies can identify associations between medications and microbiome changes, they often fall short in providing mechanistic insights. A significant limitation of the current body of literature is the lack of studies involving treatment-naïve patients, which constrains our ability to fully elucidate the specific effects of psychotropic drugs on the gut microbiota and their role in BP treatment outcomes. Many of the studies included in this review involve participants already undergoing pharmacological treatment at the time of microbiome analysis. For example, the cross-sectional study by Aizawa *et al*. compares the gut microbiomes of individuals with BP to HCs; however, most of the patients in this study were already on various medications prior to the evaluation [134]. This factor introduces a critical caveat, as the influence of psychotropic drugs on the gut microbiome may confound the observed results, making it difficult to isolate the effects of the underlying disorder from those induced by pharmacological treatment. This gap in the literature underscores the need for studies that specifically focus on treatment-naïve individuals, which would allow for a clearer understanding of how psychotropic drugs impact the gut microbiota independent of prior treatments. Unfortunately, such data are currently lacking, and as a result, much of the existing evidence is limited in its ability to address the key question of how psychotropic drugs influence the gut microbiome and, by extension, treatment outcomes in BP. Including studies that do not focus on treatment-naïve patients poses some limitations to the interpretations and conclusions drawn from our data. We emphasize that future research should prioritize longitudinal studies that follow treatment-naïve patients to more accurately assess the relationship between psychotropic drugs, gut microbiota and BP. Such studies would provide more robust data and help refine our understanding of these complex interactions. Without experimental manipulation, it is difficult to determine whether observed microbiome shifts directly affect the drug, are a consequence of host metabolism or are mediated through immune or hormonal pathways.

While more controlled studies are essential to disentangle these effects, conducting such research in this population is inherently challenging. The vulnerability of individuals with mood disorders, coupled with the challenges of achieving strict control over medication usage in a real-world setting, makes it challenging to implement randomized controlled trials or other experimental designs. As a result, the data available are often derived from observational studies, which, while valuable, are limited in their ability to establish causality. This highlights the need for innovative study designs to address these complexities while respecting the ethical and practical constraints inherent in working with vulnerable populations.

### Future directions

The intestinal microbiome may govern varying drug responses between the sexes. An increasing number of microbiome studies have revealed endocrine bacteria that can produce and respond to hormones (e.g. 5-HT, DA and oestrogen) and regulate hormonal homeostasis by inhibiting gene transcription (e.g. prolactin) [216]. Studies have also shown that females appear to have significantly higher levels of *Bifidobacteria* and *Bilophila* than males [217,218]. This difference in baseline microbiome composition may explain why females under AAP treatment may express decreased gut microbiome species diversity (i.e. *Akkermansia muciniphila*) compared with HC, while males showed no difference [113]. Some other studies have reported associations between the menstrual cycle and BP, particularly in a subgroup of females with enhanced hormonal sensitivity. This population seems to experience heterogeneous menstrual cycle effects on depressive, hypomanic and manic episodes [219], leading to greater chronobiological disruptions across the follicular and luteal phases [220]. Sex-biassed responses to psychotropics can also be due to inherent differences in physiology between sexes. Compared with females, on average, males have a higher dissolution rate, which is the rate-limiting step in drug absorption, as well as larger fluid volumes in the stomach and small intestine (following the normalization of body weight) [221]. Females, on the other hand, have, on average, higher fasted state pH, lower acid secretion, smaller stomachs and longer colonic transit time [222,224]. These can all influence the ionization and solubility of pH-sensitive drugs, thereby affecting absorption and drug bioavailability [225,226]. Notably, pre-menopausal females have a significantly longer gastric emptying time, while post-menopausal females have a shorter time, which is more similar to average males [227].

Metabolic dysfunction has been one of the most common side effects of psychotropics [228,229], along with obesity [230] and appetite dysregulation [231,232]. In a recent meta-analysis, all of the antidepressants tested, including mirtazapine, duloxetine, sertraline, venlafaxine, citalopram, fluoxetine, escitalopram, trazodone, amitriptyline, paroxetine, nortriptyline and dosulepin, increased weight gain in a population cohort [233]. Sertraline, fluoxetine, paroxetine and fluvoxamine persist in the ileum and colon at sufficient concentrations for durations long enough to directly impact the gut microbiome of both regions [234]. Serotonergic neurons, the target of some of these SSRIs, are involved in regulating gut motility, so they can explain the common adverse effects such as nausea, constipation or diarrhoea [235,236]. Antipsychotics, such as olanzapine and risperidone, are also known to induce significant weight gain [86,87, 89, 119]. Long-term use of olanzapine and clozapine can also cause metabolic syndrome, weight gain, obesity, type 2 diabetes, dyslipidaemia and cardiovascular disease [237].

The precise mechanism that regulates the connection between psychotropics and negative metabolic effects remains unclear, yet multiple theories have been suggested. Antipsychotics can alter the pH of the gastrointestinal tract, change mucosal integrity and permeability and interfere with bile salt production [238,240]. Even when antipsychotics are given intramuscularly or intravenously, molecules circulating in the bloodstream are still secreted back from the liver via the bile duct and into the gastrointestinal lumen, thus affecting the gut microbiome [187,241]. The effects, however, are significantly lessened this way [175,242]. Antipsychotics can also increase adipocyte lipogenesis, low-density lipoprotein cholesterol and hepatic fat content [243]. Moreover, antipsychotics can stimulate appetite and increase energy intake while decreasing energy expenditure via their sedative effect [238,240].

As discussed previously [107] and shown in the studies, many antipsychotics and antidepressants exhibit bactericidal and bacteriostatic activity and induce gut microbial antibiotic resistance [93,241, 242, 244]. For instance, sertraline, fluoxetine and paroxetine have the most antimicrobial effect, followed by fluvoxamine, escitalopram and citalopram [234]. Antipsychotics chlorpromazine, thioridazine and flupenthixol are potent antibiotics. Second- and third-generation antipsychotics, including clozapine, risperidone, olanzapine and aripiprazole, have some but not as strong antibiotic effects [245]. Co-administration of antibiotics reduced the amount of overall weight gain and peri-uterine fat accretion seen with olanzapine treatment without altering food intake or faecal output [88]. Given that antipsychotics are taken daily over long periods of time and some antibiotics have been suggested as conjunctive therapies for BP, including tetracyclines [246], such as minocycline [247,249] and doxycycline [250,252], dangers from the global spread of antibiotic resistance should be considered. Patients on antipsychotics frequently receive antidepressants and anti-inflammatory medications, which have also demonstrated antimicrobial properties [253,254]. As the gut microbiome can have significant effects on emotions and behaviour, chronic psychotropic use and linked metabolic side effects can precipitate psychiatric symptoms and suicidal behaviour [76,76, 255]. Accordingly, more caution should be made when prescribing psychotropics, which perhaps can be tapered off with alternative treatments such as cognitive behavioural therapies, education and support groups [256,257].

It has not yet been possible to establish a healthy gut composition in individuals with BP, which can be defined by a balanced and diverse microbiome, strong intestinal integrity and efficient digestive function; thus, the interindividual gastrointestinal microbiome variations further complicate effective interventions [258,259]. Some studies have demonstrated that the gut microbiota of depressed and bipolar patients has an abnormal composition [15,260, 261]. This implies that the gastrointestinal effects of psychotropics could play a role in their mechanisms of action, though further investigation is needed to validate this. Dietary interventions may help enhance responses to drug therapy [262,263] and address metabolic side effects caused by the medications [86,89]. Antioxidants and vitamin B supplements have been considered as adjuncts to antipsychotic drugs for SCZ patients [264], and high fibre intakes are correlated with reduced risk of depressive symptoms [265,266]. Furthermore, compared with non-vegetarians, vegetarians typically have a more rapid gastrointestinal transit time and gastric emptying [267]. One of the most widely studied dietary interventions is the Mediterranean Diet, characterized by a high fibre level, polyunsaturated fatty acids and anti-inflammatory and antioxidative products [268]. These can increase *Faecalibacterium* strains (linked to butyrate metabolism), diminishing gut inflammation [269]*,* reducing *Firmicutes* and *Proteobacteria* phyla [270] and stimulating fermentation in the lumen and adsorption of bile acid [267]; therefore, this diet leads to better drug adsorption and bioavailability [92]. Administering purified prebiotic Bimuno™ galacto-oligosaccharide powder with olanzapine can increase *Bifidobacterium* and attenuated weight gain induced by the AAP [86]. Similarly, administering olanzapine with an antibiotic containing neomycin, metronidazole and polymyxin B can attenuate weight gain and metabolic problems in rats [88].

Pro- and prebiotics have been used to reduce inflammation, decrease cardiovascular disease risks and promote weight loss [271,272]. They have also exhibited anxiolytic and antidepressant effects. In fact, a study of 80 first-episode drug-naive BP patients who underwent psychotropic therapy supplemented with probiotics showed clinical symptom improvements and reduced oxidative stress markers (allantoic acid, choline, creatine, hypoxanthine, inosine and uric acid) 3 months post-intervention [273]. In another research report, probiotic supplementation improved attention, psychomotor processing speed and executive functions also in 20 euthymic BP patients after 3 months [274].

Another innovative approach targeting gut microbiota in BP is FMT. This method involves transferring stool from a healthy donor into the pathological colon to restore a healthy gut microbiome and ameliorate symptoms [275]. Studies on these in the psychiatry field are still limited [276]. One significant success was reported in a case study where a BP patient, 6 months after undergoing FMT from their spouse, showed neither manic nor depressive symptoms [277]. This method also entails some complicated challenges regarding donors’ and recipients’ compatibility as well as sources [278]. In addition to documenting the detrimental effects of certain psychotropics, preclinical studies have explored strategies to counteract microbiota disturbances. Co-administration of probiotics, prebiotics or synbiotics has shown promise in attenuating weight gain and restoring microbial balance in animals treated with antipsychotics. Emerging work has also investigated the use of FMT as a tool to reverse drug-induced dysbiosis, offering a novel therapeutic adjunct. These findings collectively reinforce the importance of gut microbiota as a modifiable target in managing BP and support the need for translational research that bridges these preclinical insights with clinical outcomes.

Lastly, there are some immune-based therapeutic strategies suggested for BP and MDD patients, which do not follow the conventional treatment approach [279]. For instance, celecoxib is a nonsteroidal anti-inflammatory drug commonly used to treat arthritis and joint pain but has been shown to be highly effective for bipolar depression in minimizing anxiety and facilitating treatment response [280]. The same results were obtained with aspirin [281]. *N*-Acetylcysteine, a medication usually used to treat acetaminophen poisoning, was also proven to mitigate gut dysbiosis in mice [282] and improve depressive symptoms in BP patients [279,283].

Together, these animal studies provide a strong rationale for future clinical investigations aimed at stratifying psychotropic medications based on their gut microbiome profiles. They also support the growing interest in developing companion microbiome-targeted interventions to prevent or mitigate existing therapies’ adverse gut-mediated effects. Ultimately, these data contribute to a paradigm shift towards considering microbiota preservation – i.e. achieving ‘gut neutrality’ – as an important objective in the long-term pharmacological management of BP.

## Conclusion

Psychotropics may influence microbial physiology, altering the intestinal microbiome in ways that can impact therapeutic efficacy. These effects are particularly relevant when developing treatments for neurological disorders, as microbial metabolism can modulate drug efficacy, either enhancing or diminishing their intended effects. Research on the psychotropic effects of medications on the gut microbiome in BP has produced mixed results, likely due to factors such as the anatomy and physiology of the gastrointestinal tract, the release and absorption profiles of the medications, polypharmacy and the limitations of current study designs. Our systematic review of 12 clinical studies highlights that lithium and quetiapine promote the growth of beneficial gut bacteria associated with intestinal health but also increase the abundance of pathogenic bacteria linked to metabolic dysfunctions. These changes are more pronounced in female patients, who demonstrate greater microbial diversity shifts following treatment. Additionally, these psychotropics are associated with an increase in multidrug antibiotic resistance within the gut microbiome, raising concerns about potential long-term health risks. Despite these findings, the overall impact of antidepressants, non-quetiapine antipsychotics and anticholinergics on the gut microbiome in BP remains speculative. Further longitudinal and mechanistic studies are needed to elucidate their precise effects and underlying pathways.

Quetiapine responders – patients with improved depressive symptoms – exhibited distinct gut microbiome profiles that were more similar to those of healthy individuals compared with non-responders. This group also demonstrated neural connectivity patterns akin to healthy subjects, suggesting a possible gut-brain axis interaction contributing to treatment efficacy. Conversely, non-responders may possess inherent gut dysbiosis that compromises treatment outcomes. The role of *Clostridium bartlettii* in BP remains unclear, with mixed findings on its abundance following quetiapine treatment. These findings underscore the dual impact of psychotropic medications on gut microbiota, highlighting both potential therapeutic benefits and risks. The increased prevalence of beneficial bacteria alongside pathogenic and antibiotic-resistant strains reflects the complexity of microbial responses to treatment. While the mechanisms driving these shifts and their causal relationships remain uncertain, this analysis offers promising directions for improving treatment outcomes in BP. Leveraging insights into gut microbiome dynamics could lead to more personalized and effective therapies, reducing side effects and addressing the diverse needs of BP patients.

## Supplementary material

10.1099/mic.0.001568 Uncited Supplementary Material 1.

## Abbreviations

AAP: atypical antipsychotic
BMI: body mass index
BP: bipolar disorder
BP-I: bipolar I disorder
BP-II: bipolar II disorder
CNS: central nervous system
DMN: default mode network
ESBL: extended-spectrum beta-lactamase
FC: functional connectivity
fMRI: functional magnetic resonance imaging
FMT: faecal microbiota transplantation
GABA: gamma-aminobutyric acid
HC: healthy control
HPA: hypothalamic-pituitary-adrenal
MDD: major depressive disorder
PET: positron emission tomography
PRISMA: Preferred Reporting Items for Systematic Reviews and Meta-Analyses
SCFA: short-chain fatty acid
SCZ: schizophrenia
SERT: serotonin transporter
SSD: SCZ spectrum disorder
SSRIs: selective serotonin reuptake inhibitors
UR: unaffected relative
