# Supplementary material for: Pharmaco-psychiatry and gut microbiome: a systematic review of effects of psychotropic drugs for bipolar disorder
Source: Microbiology (Reading). 2025 Jun 18;171(6):001568. doi: 10.1099/mic.0.001568 (PMC12282230; doi:10.1099/mic.0.001568)
Supplement: Uncited Supplementary Material 1. [file mic-171-01568-s001.pdf]

SUPPLEMENTARY MATERIAL

Supplementary Figure 1. GRADE certainty of evidence assessment. Findings were created using GRADE Working Group's software GRADEpro GDT template.

Psychotropics compared to non-psychotropics for bipolar disorder: Correlation between microbial species and psychotropics

Patient or population: bipolar disorder
Setting: Inpatient, outpatient, and community
Intervention: Psychotropics
Comparison: Non-psychotropics

Outcomes	№ of participants (studies)	Study design	Intervention (n)	Certainty of the evidence (GRADE)	Findings
**Olanzapine v. Klebsiella** (*Ioannou et al., 2024*)<br>assessed with: Metagenomic shotgun sequencing	103<br>(1 non-randomised study)	cross-sectional	2.4	⊕⊕⊕◯<br>Moderate*	Positive correlation<br>beta = 0.07, SE = 0.02, p = 0.01
**Lithium v. Anaeromassilibacillus** (*Ioannou et al., 2024*)<br>assessed with: Metagenomic shotgun sequencing	103<br>(1 non-randomised study)	cross-sectional	38	⊕⊕⊕⊕<br>High	Positive correlation<br>beta = 1.28, SE = 0.30, p = 0.005
**Atypical antipsychotics v. Lachnospiraceae** (*Flowers et al., 2017*)<br>assessed with: 16S ribosomal RNA sequencing	117<br>(1 non-randomised study)	cross-sectional	49	⊕⊕⊕◯<br>Low**	Positive correlation<br>p = 0.001
**Atypical antipsychotics v. Akkermansia** (*Flowers et al., 2017*)<br>assessed with: 16S ribosomal RNA sequencing	117<br>(1 non-randomised study)	cross-sectional	49	⊕⊕⊕◯<br>Low**	Negative correlation<br>p = 0.03
**Quetiapine v. Clostridium bartlettii** (*Xi et al., 2023*)<br>assessed with: Metagenomic sequencing<br>follow-up: 4 weeks	75<br>(1 non-randomised study)	before-after	43	⊕⊕◯◯<br>Low***	Negative correlation<br>Statistics not reported
**Quetiapine v. Bacteroides sp_2_1_22** (*Xi et al., 2023*)<br>assessed with: Metagenomic sequencing<br>follow-up: mean 4 weeks	75<br>(1 non-randomised study)	before-after	43	⊕⊕◯◯<br>Low***	Negative correlation<br>Statistics not reported
**Quetiapine v. Bacteroides sp_3_1_19** (*Xi et al., 2023*)<br>assessed with: Metagenomic sequencing<br>follow-up: 4 weeks	75<br>(1 non-randomised study)	before-after	43	⊕⊕◯◯<br>Low***	Negative correlation<br>Statistics not reported
**Quetiapine v. Eubacterium biforme** (*Xi et al., 2023*)<br>assessed with: Metagenomic sequencing<br>follow-up: 4 weeks	75<br>(1 non-randomised study)	before-after	43	⊕⊕◯◯<br>Low***	Positive correlation<br>Statistics not reported
**Quetiapine v. Weissella confusa** (*Xi et al., 2023*)<br>assessed with: Metagenomic sequencing<br>follow-up: 4 weeks	75<br>(1 non-randomised study)	before-after	43	⊕⊕◯◯<br>Low***	Positive correlation<br>Statistics not reported
**Quetiapine v. Oribacterium sinus** (*Xi et al., 2023*)<br>assessed with: Metagenomic sequencing<br>follow-up: 4 weeks	75<br>(1 non-randomised study)	before-after	43	⊕⊕◯◯<br>Low***	Positive correlation<br>Statistics not reported
**Quetiapine v. Barnesiella intestinihominis** (*Xi et al., 2023*)<br>assessed with: Metagenomic sequencing<br>follow-up: 4 weeks	75<br>(1 non-randomised study)	before-after	43	⊕⊕◯◯<br>Low***	Positive correlation<br>Statistics not reported
**Quetiapine v. Bifidobacterium dentium** (*Lai et al., 2022*)<br>assessed with: Metagenomic sequencing<br>follow-up: 4 weeks	62<br>(1 non-randomised study)	before-after	62	⊕⊕◯◯<br>Low***	Negative correlation<br>Statistics not reported
**Quetiapine v. Enterococcus hirae** (*Lai et al., 2022*)<br>assessed with: Metagenomic sequencing<br>follow-up: 4 weeks	62<br>(1 non-randomised study)	before-after	62	⊕⊕◯◯<br>Low***	Negative correlation<br>Statistics not reported
**Quetiapine v. Anaerofustis stercorihominis** (*Lai et al., 2022*)<br>assessed with: Metagenomic sequencing<br>follow-up: 4 weeks	62<br>(1 non-randomised study)	before-after	62	⊕⊕◯◯<br>Low***	Positive correlation<br>Statistics not reported
**Quetiapine v. Streptococus cristatus** (*Lai et al., 2022*)<br>assessed with: Metagenomic sequencing<br>follow-up: 4 weeks	62<br>(1 non-randomised study)	before-after	62	⊕⊕◯◯<br>Low***	Positive correlation<br>Statistics not reported
**Quetiapine v. Campylobacter hominis** (*Lai et al., 2022*)<br>assessed with: Metagenomic sequencing<br>follow-up: 4 weeks	62<br>(1 non-randomised study)	before-after	62	⊕⊕◯◯<br>Low***	Positive correlation<br>Statistics not reported
**Quetiapine v. Porphyromonas uenonis** (*Lai et al., 2022*)<br>assessed with: Metagenomic sequencing<br>follow-up: 4 weeks	62<br>(1 non-randomised study)	before-after	62	⊕⊕◯◯<br>Low***	Positive correlation<br>Statistics not reported
**Quetiapine v. Anaerococcus vaginalis** (*Lai et al., 2022*)<br>assessed with: Metagenomic sequencing<br>follow-up: 4 weeks	62<br>(1 non-randomised study)	before-after	62	⊕⊕◯◯<br>Low***	Positive correlation<br>Statistics not reported
**Quetiapine v. Clostridium perfringens** (*Lai et al., 2022*)<br>assessed with: Metagenomic sequencing<br>follow-up: 4 weeks	62<br>(1 non-randomised study)	before-after	62	⊕⊕◯◯<br>Low***	Positive correlation<br>Statistics not reported
**Quetiapine v. Streptococcus parasanguinis** (*Lai et al., 2022*)<br>assessed with: Metagenomic sequencing<br>follow-up: 4 weeks	62<br>(1 non-randomised study)	before-after	62	⊕⊕◯◯<br>Low***	Positive correlation<br>Statistics not reported
**Quetiapine v. Costridium scindens** (*Lai et al., 2022*)<br>assessed with: Metagenomic sequencing<br>follow-up: 4 weeks	62<br>(1 non-randomised study)	before-after	62	⊕⊕◯◯<br>Low***	Positive correlation<br>Statistics not reported
**Quetiapine v. Streptococcus salivarius** (*Lai et al., 2022*)<br>assessed with: Metagenomic sequencing<br>follow-up: 4 weeks	62<br>(1 non-randomised study)	before-after	62	⊕⊕◯◯<br>Low***	Positive correlation<br>Statistics not reported
**Quetiapine v. Ruminococcus obeum** (*Lai et al., 2022*)<br>assessed with: Metagenomic sequencing<br>follow-up: 4 weeks	62<br>(1 non-randomised study)	before-after	62	⊕⊕◯◯<br>Low***	Positive correlation<br>Statistics not reported
**Quetiapine v. Enterobacter cloacae** (*Lai et al., 2022*)<br>assessed with: Metagenomic sequencing<br>follow-up: 4 weeks	62<br>(1 non-randomised study)	before-after	62	⊕⊕◯◯<br>Low***	Positive correlation<br>Statistics not reported
**Quetiapine v. Eubacterium rectale** (*Lu et al.2019*)<br>assessed with: qPCR<br>follow-up: 4 weeks	36<br>(1 non-randomised study)	before-after	36	⊕⊕⊕⊕<br>High	Positive correlation<br>p = 0.004

*Serious imprecision. 2.4 olanzapine equivalent users reported for 103 participants do not meet the optimal information size as calculated.
**Some concerns of risk of bias due to missing statistic details, some uncontrolled confounding factors, and inconsistency in data reporting.
***Some inconsistency in follow-up measurement. High risk of bias due to lack of statistics and uncontrolled confounding factors.

GRADE Working Group grades of evidence
High certainty: we are very confident that the true effect lies close to that of the estimate of the effect.
Moderate certainty: we are moderately confident in the effect estimate: the true effect is likely to be close to the estimate of the effect, but there is a possibility that it is substantially different.
Low certainty: our confidence in the effect estimate is limited: the true effect may be substantially different from the estimate of the effect.
Very low certainty: we have very little confidence in the effect estimate: the true effect is likely to be substantially different from the estimate of effect.

Supplementary Table 1. Database search queries.

Date	Database	Return
1946 to 2024 August 13	**Ovid MEDLINE(R) ALL**	
	1 "bipolar and related disorders"/ or bipolar disorder/	46779
	2 (bipolar or manic depress* or mania).mp.	99622
	3 1 or 2	99622
	4 gastrointestinal microbiome/ or ((gastrointestinal or intestin* or gut or bowel* or digesti* or colon or colonic or feces or faeces or fecal or faecal) and (flora or microb* or microflora or metagenom* or bacteri* or coloniz* or colonis*)).mp.	304593
	5 metagenome/ or exp bacteria/ or exp microbiota/	1644121
	6 gastrointestinal tract/ or exp intestines/ or exp lower gastrointestinal tract/	454703
	7 4 or (5 and 6)	310797
	8 antimanic agents/ or carbamazepine/ or gabapentin/ or lithium carbonate/ or lithium chloride/ or lithium compounds/ or valproic acid / or antipsychotic agents/	99118
	9 (Mood stabilizer* or mood stabiliser* or antipsychotic* or anti-psychotic* or anti-manic or antimanic or haloperidol or haldol or loxapine or loxitane or loxapine or adasuve or Aripiprazole or Abilify or Asenapine or Saphris or Cariprazine or Vraylar or Lumateperone or Caplyta or Lurasidone or Latuda or Olanzapine or Zyprexa or Olanzapine or samidorphan or Lybalvi or Quetiapine or Seroquel or risperidone or Risperdal or Ziprasidone or Geodon or Lithium or Divalproex or Valproic acid or Valproate or Carbamazepine or Oxcarbazepine or Lamotrigine or Fluoxetine or Symbyax or gabapentin).mp.	237786
	10 8 or 9	237786
	11 3 and 7 and 10	26
1806 to April Week 2 2024	**APA PsycInfo**	
	1 exp bipolar disorder/	36298
	2 (bipolar or manic depress* or mania).mp.	63150
	3 1 or 2	63343
	4 (gastrointestinal or intestin* or gut or bowel* or digesti* or colon or colonic or feces or faeces or fecal or faecal) and (flora or microb* or microflora or metagenom* or bacteri* or coloniz* or colonis*)).mp.	3465
	5 exp Valproic Acid / or exp Mood Stabilizers/ or exp Carbamazepine/ or exp Lithium/	10783
	6 (Mood stabilizer* or mood stabiliser* or antipsychotic* or anti-psychotic* or anti-manic or antimanic or haloperidol or haldol or loxapine or loxitane or loxapine or adasuve or Aripiprazole or Abilify or Asenapine or Saphris or Cariprazine or Vraylar or Lumateperone or Caplyta or Lurasidone or Latuda or Olanzapine or Zyprexa or Olanzapine or samidorphan or Lybalvi or Quetiapine or Seroquel or risperidone or Risperdal or Ziprasidone or Geodon or Lithium or Divalproex or Valproic acid or Valproate or Carbamazepine or Oxcarbazepine or Lamotrigine or Fluoxetine or Symbyax or gabapentin).mp.	81284
	7 5 or 6	81284
	8 3 and 4 and 7	12
	**SCOPUS (Advanced search)**	
	TITLE-ABS-KEY(bipolar or manic-depress* or mania) AND TITLE-ABS-KEY((gastrointestinal or intestin* or gut or bowel* or digesti* or colon or colonic or feces or faeces or fecal or faecal) and (flora or microb* or microflora or metagenom* or bacteri* or coloniz* or colonis*)) AND TITLE-ABS-KEY(Mood-stabilizer* or mood-stabiliser* or antipsychotic* or anti-psychotic* or anti-manic or antimanic or haloperidol or haldol or loxapine or loxitane or loxapine or adasuve or Aripiprazole or Abilify or Asenapine or Saphris or Cariprazine or Vraylar or Lumateperone or Caplyta or Lurasidone or Latuda or Olanzapine or Zyprexa or Olanzapine or samidorphan or Lybalvi or Quetiapine or Seroquel or risperidone or Risperdal or Ziprasidone or Geodon or Lithium or Divalproex or Valproic-acid or Valproate or Carbamazepine or Oxcarbazepine or Lamotrigine or Fluoxetine or Symbyax or gabapentin)	123
1974 to 2024 August 13	**Embase**	
	1 exp bipolar disorder/	86071
	2 (bipolar or manic depress* or mania).mp.	161200
	3 1 or2	161512
	4 exp intestine flora/	120047
	5 microflora/ or feces microflora/	52160
	6 exp intestine/	542783
	7 exp gastrointestinal tract/	82052
	8 5 and (6 or 7)	9469
	9 (gastrointestinal or intestin* or gut or bowel* or digesti* or colon or colonic or feces or faeces or fecal or faecal) and (flora or microb* or microflora or metagenom* or bacteri* or coloniz* or colonis*)).mp.	379936
	10 4 or 8 or 9	380074
	11 exp mood stabilizer/	150399
	12 (Mood stabilizer* or mood stabiliser* or antipsychotic* or anti-psychotic* or anti-manic or antimanic or haloperidol or haldol or loxapine or loxitane or loxapine or adasuve or Aripiprazole or Abilify or Asenapine or Saphris or Cariprazine or Vraylar or Lumateperone or Caplyta or Lurasidone or Latuda or Olanzapine or Zyprexa or Olanzapine or samidorphan or Lybalvi or Quetiapine or Seroquel or risperidone or Risperdal or Ziprasidone or Geodon or Lithium or Divalproex or Valproic acid or Valproate or Carbamazepine or Oxcarbazepine or Lamotrigine or Fluoxetine or Symbyax or gabapentin).mp.	457304
	13 11 or 12	457490
	14 3 and 10 and 13	137

13 August 2024	**PubMed**	
	Search: ((bipolar[Title/Abstract] OR manic-depress*[Title/Abstract] OR mania[Title/Abstract]) AND (gastrointestinal[Title/Abstract] OR intestin*[Title/Abstract] OR gut[Title/Abstract] OR bowel*[Title/Abstract] OR digesti*[Title/Abstract] OR colon[Title/Abstract] OR colonic[Title/Abstract] OR feces[Title/Abstract] OR faeces[Title/Abstract] OR fecal[Title/Abstract] OR faecal[Title/Abstract])) AND (Mood-stabilizer*[Title/Abstract] OR mood-stabiliser*[Title/Abstract] OR antipsychotic*[Title/Abstract] OR anti-psychotic*[Title/Abstract] OR anti-manic[Title/Abstract] OR antimanic[Title/Abstract] OR haloperidol[Title/Abstract] OR haldol[Title/Abstract] OR loxapine[Title/Abstract] OR loxitane[Title/Abstract] OR loxapine[Title/Abstract] OR adasuve[Title/Abstract] OR Aripiprazole[Title/Abstract] OR Abilify[Title/Abstract] OR Asenapine[Title/Abstract] OR Saphris[Title/Abstract] OR Cariprazine[Title/Abstract] OR Vraylar[Title/Abstract] OR Lumateperone[Title/Abstract] OR Caplyta[Title/Abstract] OR Lurasidone[Title/Abstract] OR Latuda[Title/Abstract] OR Olanzapine[Title/Abstract] OR Zyprexa[Title/Abstract] OR Olanzapine[Title/Abstract] OR samidorphan[Title/Abstract] OR Lybalvi[Title/Abstract] OR Quetiapine[Title/Abstract] OR Seroquel[Title/Abstract] OR risperidone[Title/Abstract] OR Risperdal[Title/Abstract] OR Ziprasidone[Title/Abstract] OR Geodon[Title/Abstract] OR Lithium[Title/Abstract] OR Divalproex[Title/Abstract] OR Valproic-acid[Title/Abstract] OR Valproate[Title/Abstract] OR Carbamazepine[Title/Abstract] OR Oxcarbazepine[Title/Abstract] OR Lamotrigine[Title/Abstract] OR Fluoxetine[Title/Abstract] OR Symbyax[Title/Abstract] OR gabapentin[Title/Abstract])	171

Supplementary Table 2. PRISMA 2020 abstract checklist. Table adapted from Page MJ, McKenzie JE, Bossuyt PM, Boutron I, Hoffmann TC, Mulrow CD, et al. The PRISMA 2020 statement: an updated guideline for reporting systematic reviews. BMJ 2021;372:n71. doi: 10.1136/bmj.n71. This work is licensed under CC BY 4.0. To view a copy of this license, visit https://creativecommons.org/licenses/by/4.0

Section and Topic	Item #	Checklist item	Reported (Yes/No)
**TITLE**			
Title	1	Identify the report as a systematic review.	Yes
**BACKGROUND**			
Objectives	2	Provide an explicit statement of the main objective(s) or question(s) the review addresses.	Yes
**METHODS**			
Eligibility criteria	3	Specify the inclusion and exclusion criteria for the review.	Yes
Information sources	4	Specify the information sources (e.g. databases, registers) used to identify studies and the date when each was last searched.	Yes
Risk of bias	5	Specify the methods used to assess risk of bias in the included studies.	Yes
Synthesis of results	6	Specify the methods used to present and synthesise results.	
**RESULTS**			
Included studies	7	Give the total number of included studies and participants and summarise relevant characteristics of studies.	Yes
Synthesis of results	8	Present results for main outcomes, preferably indicating the number of included studies and participants for each. If meta-analysis was done, report the summary estimate and confidence/credible interval. If comparing groups, indicate the direction of the effect (i.e. which group is favoured).	Yes
**DISCUSSION**			
Limitations of evidence	9	Provide a brief summary of the limitations of the evidence included in the review (e.g. study risk of bias, inconsistency and imprecision).	Yes
Interpretation	10	Provide a general interpretation of the results and important implications.	Yes
**OTHER**			
Funding	11	Specify the primary source of funding for the review.	No
Registration	12	Provide the register name and registration number.	Yes

Supplementary Table 3. PRISMA 2020 checklist. Table adapted from Page MJ, McKenzie JE, Bossuyt PM, Boutron I, Hoffmann TC, Mulrow CD, et al. The PRISMA 2020 statement: an updated guideline for reporting systematic reviews. BMJ 2021;372:n71. doi: 10.1136/bmj.n71. This work is licensed under CC BY 4.0. To view a copy of this license, visit https://creativecommons.org/licenses/by/4.0/

Section and Topic	Item #	Checklist item	Location (page #)
**TITLE**			
Title	1	Identify the report as a systematic review.	1
**ABSTRACT**			
Abstract	2	See the PRISMA 2020 for Abstracts checklist.	1-2
**INTRODUCTION**			
Rationale	3	Describe the rationale for the review in the context of existing knowledge.	7
Objectives	4	Provide an explicit statement of the objective(s) or question(s) the review addresses.	7
**METHODS**			
Eligibility criteria	5	Specify the inclusion and exclusion criteria for the review and how studies were grouped for the syntheses.	7
Information sources	6	Specify all databases, registers, websites, organizations', reference lists and other sources searched or consulted to identify studies. Specify the date when each source was last searched or consulted.	8<br>S1 Table
Search strategy	7	Present the full search strategies for all databases, registers and websites, including any filters and limits used.	8<br>S1 Table
Selection process	8	Specify the methods used to decide whether a study met the inclusion criteria of the review, including how many reviewers screened each record and each report retrieved, whether they worked independently, and if applicable, details of automation tools used in the process.	7
Data collection process	9	Specify the methods used to collect data from reports, including how many reviewers collected data from each report, whether they worked independently, any processes for obtaining or confirming data from study investigators, and if applicable, details of automation tools used in the process.	8
Data items	10a	List and define all outcomes for which data were sought. Specify whether all results that were compatible with each outcome domain in each study were sought (e.g. for all measures, time points, analyses), and if not, the methods used to decide which results to collect.	8
	10b	List and define all other variables for which data were sought (e.g. participant and intervention characteristics, funding sources). Describe any assumptions made about any missing or unclear information.	8
Study risk of bias assessment	11	Specify the methods used to assess risk of bias in the included studies, including details of the tool(s) used, how many reviewers assessed each study and whether they worked independently, and if applicable, details of automation tools used in the process.	8-9
Effect measures	12	Specify for each outcome the effect measure(s) (e.g. risk ratio, mean difference) used in the synthesis or presentation of results.	S2 Table
Synthesis methods	13a	Describe the processes used to decide which studies were eligible for each synthesis (e.g. tabulating the study intervention characteristics and comparing against the planned groups for each synthesis (item #5)).	8
	13b	Describe any methods required to prepare the data for presentation or synthesis, such as handling of missing summary statistics, or data conversions.	8
	13c	Describe any methods used to tabulate or visually display results of individual studies and syntheses.	8
	13d	Describe any methods used to synthesize results and provide a rationale for the choice(s). If meta-analysis was performed, describe the model(s), method(s) to identify the presence and extent of statistical heterogeneity, and software package(s) used.	8
	13e	Describe any methods used to explore possible causes of heterogeneity among study results (e.g. subgroup analysis, meta-regression).	8
	13f	Describe any sensitivity analyses conducted to assess robustness of the synthesized results.	N/A

Section and Topic	Item #	Checklist item	Location (page #)
Reporting bias assessment	14	Describe any methods used to assess risk of bias due to missing results in a synthesis (arising from reporting biases).	8-9
Certainty assessment	15	Describe any methods used to assess certainty (or confidence) in the body of evidence for an outcome.	8-9
**RESULTS**			
Study selection	16a	Describe the results of the search and selection process, from the number of records identified in the search to the number of studies included in the review, ideally using a flow diagram.	Fig 1
	16b	Cite studies that might appear to meet the inclusion criteria, but which were excluded, and explain why they were excluded.	N/A
Study characteristics	17	Cite each included study and present its characteristics.	9-11<br>Table 1, 2
Risk of bias in studies	18	Present assessments of risk of bias for each included study.	Fig 2
Results of individual studies	19	For all outcomes, present, for each study: (a) summary statistics for each group (where appropriate) and (b) an effect estimate and its precision (e.g. confidence/credible interval), ideally using structured tables or plots.	Table 3-6
Results of syntheses	20a	For each synthesis, briefly summarize the characteristics and risk of bias among contributing studies.	Supp Fig 1
	20b	Present results of all statistical syntheses conducted. If meta-analysis was done, present for each the summary estimate and its precision (e.g. confidence/credible interval) and measures of statistical heterogeneity. If comparing groups, describe the direction of the effect.	N/A
	20c	Present results of all investigations of possible causes of heterogeneity among study results.	9-12
	20d	Present results of all sensitivity analyses conducted to assess the robustness of the synthesized results.	N/A
Reporting biases	21	Present assessments of risk of bias due to missing results (arising from reporting biases) for each synthesis assessed.	Fig 2
Certainty of evidence	22	Present assessments of certainty (or confidence) in the body of evidence for each outcome assessed.	Supp Fig 1
**DISCUSSION**			
Discussion	23a	Provide a general interpretation of the results in the context of other evidence.	24-25
	23b	Discuss any limitations of the evidence included in the review.	26-29
	23c	Discuss any limitations of the review processes used.	25
	23d	Discuss implications of the results for practice, policy, and future research.	35-39
**OTHER INFORMATION**			
Registration and protocol	24a	Provide registration information for the review, including register name and registration number, or state that the review was not registered.	2<br>Editorial manager
	24b	Indicate where the review protocol can be accessed, or state that a protocol was not prepared.	40, 43-47<br>Editorial manager

Section and Topic	Item #	Checklist item	Location (page #)
	24c	Describe and explain any amendments to information provided at registration or in the protocol.	Editorial manager
Support	25	Describe sources of financial or non-financial support for the review, and the role of the funders or sponsors in the review.	40
Competing interests	26	Declare any competing interests of review authors.	40
Availability of data, code and other materials	27	Report which of the following are publicly available and where they can be found: template data collection forms; data extracted from included studies; data used for all analyses; analytic code; any other materials used in the review.	40-47
